# Stromal peroxidasin drives early tumor growth in breast cancer

**DOI:** 10.1016/j.isci.2026.116078

**Published:** 2026-06-01

**Authors:** Kaitlin Wyllie, Ellie T.Y. Mok, Que Emmi Tran, Elysse C. Filipe, Jessica L. Chitty, Ron Enriquez, Anaiis Zaratzian, Andrew M. Da Silva, Michael Tayao, David Gallego-Ortega, Sandra O’Toole, Amelia L. Parker, Vasilios Panagopoulos, Thomas R. Cox

**Affiliations:** 1 The Garvan Institute of Medical Research and The Kinghorn Cancer Centre, Sydney, NSW, Australia; 2 School of Clinical Medicine, St Vincent’s Healthcare Clinical Campus, Faculty of Medicine and Health, UNSW, Sydney, NSW, Australia; 3 School of Biomedical Engineering, University of Technology, Sydney, NSW, Australia; 4 Department of Tissue Pathology and Diagnostic Oncology, Royal Prince Alfred Hospital and NSW Health Pathology, Sydney, NSW, Australia; 5 Centre for Cancer Biology, College of Health, Adelaide University, Adelaide, SA, Australia

**Keywords:** molecular biology, cell biology, cancer systems biology

## Abstract

The extracellular matrix (ECM) plays multifaceted tumor-promoting and tumor-restraining roles in breast cancer progression. Extracellular peroxidases represent a critical yet underexplored component of stromal remodeling machinery. Peroxidasin (PXDN), an extracellular peroxidase with established roles in collagen IV crosslinking, has been associated with poor outcomes in several cancers, but its role in breast cancer remains unclear. Through temporal proteomic analysis of dysregulated ECM proteins, we identified PXDN as upregulated during early tumor development in mouse models. In human breast cancers, PXDN expression shows compartment-specific associations with patient outcome, with high stromal PXDN, predominantly cancer-associated fibroblast (CAF)-derived, correlating with poor prognosis. We demonstrate that PXDN regulates CAF behavior, with subsequent matrix remodeling affecting cancer cell behavior. Reduction of CAF-derived PXDN *in vivo* slows tumor development, while pharmacological inhibition of extracellular peroxidases improves overall survival. These findings establish PXDN as a mediator of breast tumor progression and a promising future therapeutic target.

## Highlights

• High stromal peroxidasin predicts poor patient survival
• Peroxidasin expression peaks during early breast cancer development
• Cancer-associated fibroblasts are a major source of tumor-promoting peroxidasin
• Targeting extracellular peroxidases extends survival in breast cancer models

## Graphical abstract

**Figure:**
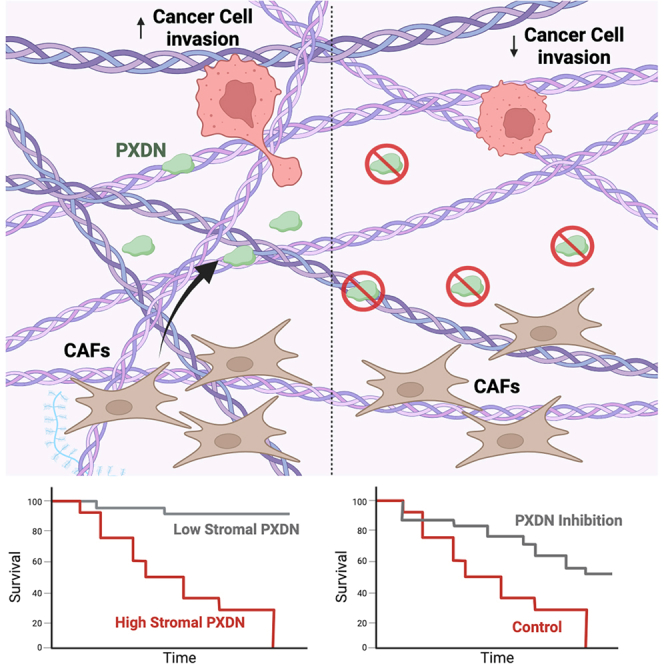

## Introduction

Breast cancer is the most prevalent cancer among women worldwide and remains the leading cause of cancer-related mortality for women.^1^^,^^2^ Despite significant advancements in early detection and rates of surgical resection over recent decades leading to relatively high 5-year survival rates for stage I and II breast cancer patients (85%–90%), this survival drops dramatically to around 29% for patients with stage IV disease.^3^ Moreover, recurrence remains a significant concern, affecting an estimated 12%–41% of patients.^4^^,^^5^^,^^6^ These statistics highlight the need to improve current treatments for these patients.

One major contributor to poor outcomes in breast cancer is thought to be the pro-tumorigenic effects of the fibrotic stroma typically found within and surrounding tumors.^7^^,^^8^^,^^9^^,^^10^^,^^11^^,^^12^ The extracellular matrix (ECM), a key component of the stroma, not only plays a complex biological role in modulating tumor growth, metastasis, and resistance to therapy,^13^ but also acts as a modulator of therapy efficacy, such as by affecting drug delivery.^14^^,^^15^^,^^16^^,^^17^ Importantly, previous studies have shown that the stroma can exert both tumor-restrictive and tumor-promoting effects, depending on its composition and context, and that these effects also change over time. For example, fibrillar collagens such as collagen I can be tumor suppressive by encapsulating tumor cells within a dense stroma,^18^ or have pro-tumorigenic effects leading to increased cell adhesion, proliferation, and migration.^19^^,^^20^ This demonstrates a need for nuanced therapeutic approaches which selectively target only the specific ECM components or their modifications that contribute to tumor progression.^21^

Among these ECM components, peroxidasin (PXDN) has emerged as a molecule of interest. PXDN is an ECM enzyme involved in facilitating the NC1 domain crosslinking of collagen IV molecules,^22^^,^^23^^,^^24^ a process essential for maintaining basement membrane integrity in healthy tissues. Importantly, in fibrotic tumors, PXDN has also been shown to be upregulated.^25^ The impact that this aberrant PXDN expression has on tumor progression is not fully understood. For instance, while high PXDN is associated with worse overall survival in mesothelioma, melanoma, glioblastoma, bladder, cervical, stomach, and thyroid cancer,^26^ no association between PXDN expression and survival was reported in breast cancer. There is, however, previous work linking PXDN expression to breast epithelial cell and mesenchymal stromal cell proliferation, migration, and epithelial-to-mesenchymal transition.^27^^,^^28^^,^^29^^,^^30^

In this study, we identify PXDN as upregulated in breast cancer progression and sought to better understand the patterns of PXDN expression in breast cancer, its potential prognostic value, role in driving cancer progression, and the potential therapeutic benefit of targeted PXDN inhibition in breast cancer.

## Results

### Peroxidasin is upregulated in early stages of tumor development in the PyMT mouse model of breast cancer

Previous studies have demonstrated that PXDN is upregulated in multiple cancer types, but the expression patterns of PXDN in breast tumors compared to healthy tissues remains less clear.^26^ We sought to gain a deeper understanding of the temporal dynamics of PXDN expression during breast cancer progression. We have previously generated a mass spectrometry dataset by collecting tumors from the PyMT genetically engineered mouse model of mammary carcinoma at early (hyperplastic), mid (adenoma/adenocarcinoma), and late (metastatic adenocarcinoma) stages of disease, alongside age-matched mammary fat pads from healthy control mice.^31^ The PyMT model is a well-established system that closely mirrors the histopathological progression observed in human breast cancer, including the transition from pre-malignant lesions to early adenoma and finally progression to metastatic adenocarcinoma.^32^^,^^33^^,^^34^ This makes it an ideal immunocompetent, and spontaneous model for investigating temporal changes in the tumor microenvironment.

**Figure 1:**
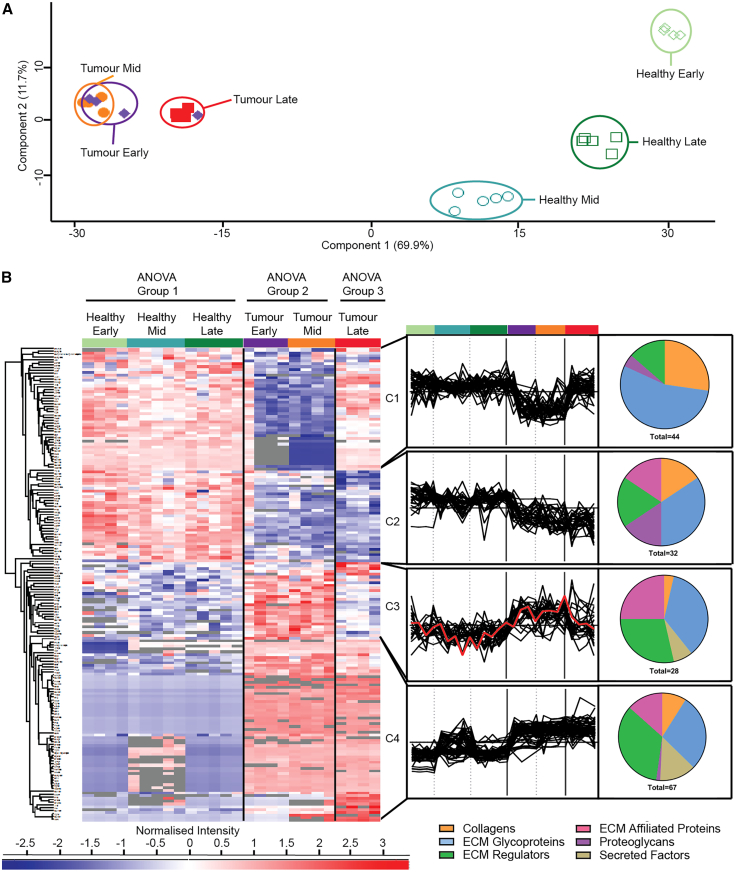
Significantly differentially expressed ECM molecules in early, mid and late-stage mammary tumor samples from the PyMT mouse model of breast cancer in comparison to healthy fat pad tissue (A) PCA of individual samples (*n* = 4 or 5 mice per group). Principal components 1 and 2 account for 81.6% of the variability in the dataset. (B) Heatmap of significantly differentially deposited matrisomal elements between groups within the dataset (left). Missing values are shaded in gray. Four major clusters were identified based on Euclidian hierarchical clustering of proteins (middle) and the matrisome categorical annotation for these clustered matrisomal elements based on the matrisome project is shown (right). Expression of PXDN in each sample (cluster 3) is highlighted in red.

Analysis of the dataset focused on identifying dysregulation of ECM in tumors compared to healthy tissue and across disease stages. A total of 4,283 proteins were robustly identified in tumor and mammary fat-pad samples. These proteins were filtered to contain only proteins known to be ECM or ECM-associated components (*n* = 247) based on the Naba Lab matrisome list.^35^ Principal component analysis (PCA) revealed that individual samples clustered according to both disease status (tumor versus healthy) and the age of the mice, which corresponded to disease stage in tumor bearing mice (Figure 1A). This indicated that the primary sources of variance (∼70%) in the dataset were the presence of tumors (vs. no tumor). Hierarchical clustering was then performed on the significantly differentially expressed proteins. Groupings for ANOVA analysis were informed by PCA clustering, with all healthy samples grouped together, all late-stage tumors samples grouped together, and all early and mid-stage tumors samples grouped together. This resulted in *n* = 171 significantly differentially deposited matrisomal proteins (Figure 1B and Table S1).

This analysis revealed four distinct expression clusters (C1–C4) (Figure 1B, middle). The first cluster (C1) included ECM molecules that were downregulated in early and mid-stage tumors compared to healthy but increased in abundance at the later stage. These were predominantly ECM glycoproteins and collagens, consistent with the literature that shows that the matrix architecture is degraded in tumors followed by aberrant ECM deposition in advanced disease.^36^^,^^37^^,^^38^^,^^39^^,^^40^ The second cluster (C2) comprised matrisomal elements that were consistently downregulated across all tumor stages. Further characterization of this group is needed to determine the potential functional role of this group in restraining tumor progression. The third cluster (C3) contained matrix molecules that were upregulated during early tumor formation but showed reduced expression in late-stage tumors. PXDN was a member of this cluster, as indicated by the red line in the profile plots (Figure 1B, middle), suggesting that its expression peaks during the early phases of tumorigenesis. The fourth cluster (C4) included matrix molecules that remained upregulated throughout all stages of tumor development. This group was enriched for ECM regulators and glycoproteins, which aligns with the persistent remodeling of the ECM observed during tumor progression.^36^^,^^37^^,^^38^^,^^39^^,^^40^

### PXDN expression at the RNA and protein level is associated with patient outcomes in breast cancer

To further investigate the potential role of PXDN in driving breast cancer progression and influencing clinical outcomes, we analyzed its association with patient survival in human datasets. This analysis aimed to determine whether PXDN expression correlates with disease severity and/or prognosis, which might indicate PXDN is playing a role in shaping disease trajectory.

**Figure 2:**
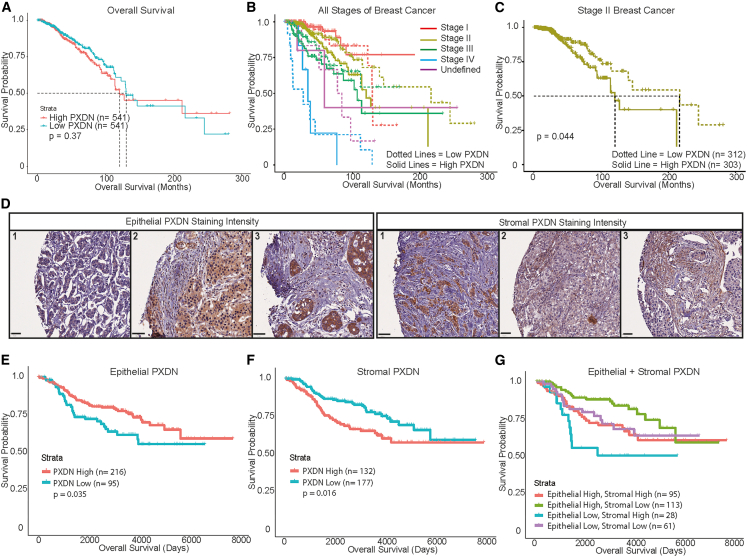
Association of PXDN expression with patient overall survival Significance between Kaplan-Meier survival curves were calculated with the log-rank test. (A) Overall survival of 1,082 patients from the TCGA breast cancer cohort stratified by median mRNA expression of PXDN. (B) Overall survival of 1,082 patients from the TCGA breast cancer cohort separated by disease stage, and stratified by median mRNA expression of PXDN (dotted line = low PXDN expression, solid line = high PXDN expression) and stage of disease (stage I, red; II, gold; III, green; IV, blue; or unknown, purple). (C) Extraction of data from (B) showing patients with stage II breast cancer at the time of diagnosis stratified by median mRNA expression of PXDN (dotted line = low PXDN expression, solid line = high PXDN expression) (*n* = 615). (D) Examples of PXDN IHC staining intensity and corresponding intensity scores for the stromal and epithelial compartments of tumors (*n* = 334). Scale bars, 50 μm. (E) Overall survival of 311 invasive ductal carcinoma patients from the CREA tumor microarray cohort stratified by high vs. low Allred-scores for PXDN IHC staining of epithelial compartments of tumors. (F) Overall survival of 309 invasive ductal carcinoma patients from the CREA tumor microarray cohort stratified by high vs. low Allred-scores for PXDN IHC staining of stromal compartments of tumors. (G) Overall survival of invasive ductal carcinoma patients from the CREA tumor microarray cohort stratified by combined stromal and epithelial Allred-scores for PXDN IHC staining of cores. Log-rank *p* values between each curve of (G) are listed in Table S3.

Using data from The Cancer Genome Atlas (TCGA) (Table S2), we assessed the relationship between PXDN mRNA expression and invasive breast cancer patient outcomes by univariate analysis using log-rank tests. When patients were stratified by median PXDN mRNA expression, no significant association was observed with overall survival (Figure 2A). These findings were validated in an independent dataset using the online Kaplan-Meier tool, which similarly showed no significant association between PXDN expression and overall survival when stratified by median expression levels (Figure S1A).

Given the higher expression of PXDN in earlier stages of disease in our mouse model data, we next investigated the association between PXDN expression and overall survival in breast cancer patients at different stages of disease. While there was no association between PXDN expression and patient overall survival in patients with stage I, III, or IV breast cancer (Figures 2B and S1B–S1D), there was an association between poor overall survival and high PXDN expression in stage II breast cancer patients (*p* = 0.044) (Figure 2C). These data support the mass spectrometry data from mouse models suggesting that PXDN may play a role in breast cancer progression and prognosis at earlier stages of disease development.

To further explore the prognostic significance of PXDN at the protein level, as well as to identify any potential spatial patterns of PXDN expression in tumors, we analyzed a tissue microarray (TMA) comprising 334 invasive ductal carcinoma samples collected from patients between 1992 and 2002 (Tables 1 and S4). Tumor sections were stained for PXDN by immunohistochemistry (IHC), and Allred scores were calculated and stratified by high vs. low expression of PXDN for epithelial and stromal compartments of the tumor separately.

**Table 1:** Demographics of patients in the high and low PXDN groups (stromal or epithelial) in the CREA TMA cohort of breast cancer

	Epithelial PXDN		*p* value	Stromal PXDN		*p* value
	Low	High		Low	High	
**Total Patients**	101	220		186	133	
**Grade**						
1	8 (7.9%)	35 (15.9%)	**0.0346**^b^	26 (14%)	19 (14.3%)	**0.0112**^b^
2	28 (27.7%)	78 (35.4%)		68 (36.6%)	38 (28.6%)	
3	54 (24.5%)	94 (42.7%)		73 (39.2%)	72 (54.1%)	
Unclassified	11 (5%)	13 (5.9%)		19 (10.2%)	4 (3%)	
**Molecular Subtype**						
Luminal A	50 (49.5%)	136 (61.8%)	**0.0367**^a^	116 (62.4%)	69 (51.9%)	0.1212^a^
Luminal B	9 (8.9%)	29 (13.2%)		17 (9.1%)	19 (14.3%)	
HER2	13 (12.9%)	13 (5.9%)		14 (7.5%)	12 (9%)	
Basal-like	19 (18.8%)	30 (13.6%)		23 (12.4%)	26 (19.5%)	
Unclassified	10 (9.9%)	12 (5.5%)		16 (8.6%)	7 (5.3%)	
**Treatment**						
None	16 (15.8%)	38 (17.3%)	0.9429^a^	31 (16.7%)	25 (18.8%)	0.1092^a^
Chemotherapy	18 (17.8%)	36 (16.4%)		31 (16.7%)	25 (18.8%)	
Endocrine therapy	10 (9.9%)	18 (8.2%)		8 (4.3%)	19 (14.3%)	
Both	20 (19.8%)	44 (20%)		35 (18.8%)	29 (21.8%)	
Information missing	37 (36.6%)	84 (38.2%)		81 (43.5%)	35 (26.3%)	

Percentages were calculated using a sum of all patients within a high or low PXDN grouping for each clinical factor.

a chi squared test for independence.

b Fishers exact test for independence. *p* values<0.05 are bolded.

In the epithelial compartment, high PXDN staining was significantly associated with better overall survival (*p* = 0.035) (Figure 2E). In contrast, high PXDN staining in the stromal compartment was significantly associated with worse overall survival (*p* = 0.016) (Figure 2F). When both stromal and epithelial PXDN scores were considered together, patients with either high epithelial and high stromal PXDN staining (red), or low stromal and low epithelial PXDN staining (purple), exhibited similar intermediate survival outcomes (Figure 2G). Notably, patients with the combination of low epithelial and high stromal PXDN expression (blue) had the poorest overall survival, while patients with high epithelial and low stromal PXDN expression (green) had the best overall survival (Figure 2G). These findings highlight the importance of compartment-specific PXDN and may explain why a stronger association with survival was observed at the protein level compared to bulk transcriptomic analysis where spatial localization is not accounted for. The data also suggest a compartment-specific role for PXDN in breast cancer progression.

Further analysis of patient clinicopathological features revealed that high epithelial PXDN levels were correlated with the Luminal A subtype of breast cancer (Table 1). In contrast, high stromal PXDN levels were associated with higher tumor grade, which corresponds to level of tumor differentiation (Table 1). The associations between PXDN and molecular subtype, and tumor grade may be linked since low grade tumors are more common in luminal subtypes of breast cancer.^41^^,^^42^ While both stromal and epithelial PXDN levels were predictive of survival in univariate cox proportional models, they were not independently predictive of patient survival in a multivariate cox proportional model including tumor grade and molecular subtype information (Table 2). Thus, at this point, PXDN alone whilst not an independent prognostic marker for breast cancer, likely plays an important biological role and remains an interesting protein to study further to better understand its contribution to breast cancer progression.

**Table 2:** Univariate and multivariate Cox proportional hazard models showing variables that are associated with patient survival in the CREA TMA cohort of breast cancer

Variables	Univariate analysis		Multivariate analysis	
	HR (95% CI)	*p* value	HR (95% CI)	*p* value
Age	1.001 (0.979, 1.024)	0.938	NA	NA
Grade				
**1**				
2	2.356 (0.693, 8.009)	0.170	2.457 (0.309, 19.541)	0.395
3	7.574 (2.371, 24.189)	**0.001**	3.943 (0.482, 32.277)	0.201
Molecular subtype				
**Luminal A**				
Luminal B	2.951 (1.525, 5.707)	**0.001**	3.354 (1.283, 8.768)	**0.014**
HER2	4.278 (2.214, 8.267)	**<0.001**	3.845 (1.482, 9.976)	**0.006**
Basal-like	3.690 (2.120, 6.422)	**<0.001**	2.467 (0.951, 6.392)	0.063
Treatment				
**None**				
Chemotherapy	0.690 (0.329, 1.444)	0.324	0.790 (0.349, 1.790)	0.573
Endocrine therapy	2.158 (1.073, 4.342)	**0.031**	1.415 (0.607, 3.300)	0.421
Both	0.676 (0.323, 1.417)	0.300	0.882 (0.398, 1.956)	0.758
Epithelial PXDN				
**Low**				
High	0.619 (0.395, 0.970)	**0.036**	1.132 (0.606, 2.114)	0.698
Stromal PXDN				
**Low**				
High	1.701 (1.099, 2.633)	**0.017**	1.164 (0.641, 2.112)	0.618

Bolded variables were used as references. Age was excluded from multivariate analysis since it was not significantly associated with survival in univariate analysis. Multivariate analysis concordance = 0.775, likelihood ratio test *p* = 0.0001, Wald test *p* = 0.0008, log-rank test *p* = 0.00002. *p* values<0.05 are bolded.

### CAFs are a major cell type responsible for stromal PXDN production in tumors

**Figure 3:**
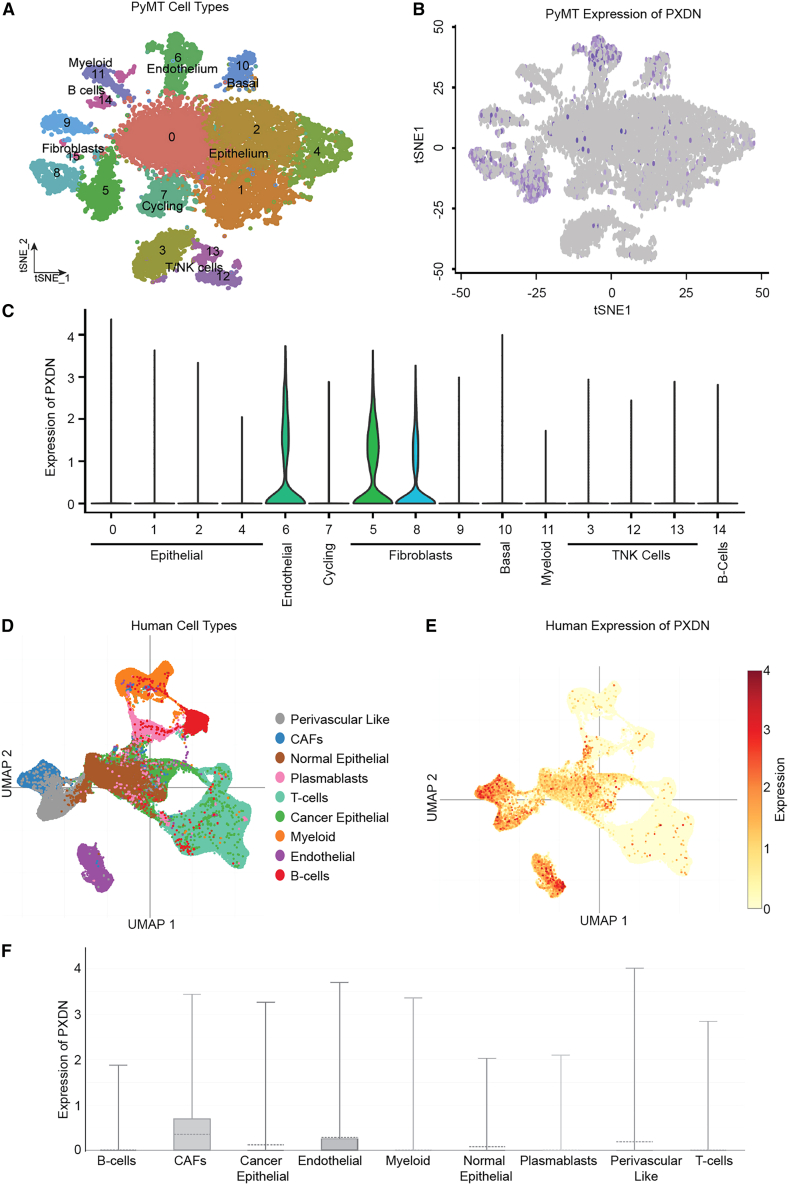
Single cell analysis of breast tumor cells expressing PXDN (A–C) Murine PyMT tumor single cell data (11,490 cells) obtained from Valdés-Mora et al.^43^ (A) UMAP of the major cell types identified in the murine single cell data. (B) UMAP of PXDN expression across cells. (C) Violin plots of fold change in PXDN expression according to cell type in the murine dataset. (D and E) Human breast cancer single cell and spatial data containing 130,246 cells from 26 invasive breast cancer patients. (D) UMAP of the major cell types identified in the human single cell data. (E) UMAP of PXDN expression across human cells. (F) Boxplots of fold change in PXDN expression according to cell type in the human dataset.

Based on the different associations of stromal and epithelial PXDN with patient survival, we sought to identify the cell types responsible for PXDN production within the tumor microenvironment. To gain insight into the cellular sources of PXDN, we analyzed existing single-cell RNA sequencing data of breast tumors from the PyMT murine model^43^ (Figure 3A). We found that PXDN was most highly expressed in endothelial cell and fibroblast populations (Figures 3B and 3C).

To further validate that CAFs may be the major source of stromal-derived PXDN in human breast cancers, a human single-cell RNA sequencing dataset was accessed via the Broad Institute’s Single Cell Portal^44^ consisting of a single cell and spatially resolved atlas of human breast cancers.^45^ This dataset included 22 invasive ductal carcinomas, two invasive lobular carcinomas, and two metastatic breast cancer patients across all major molecular subtypes of breast cancer from which 130,246 cells were used to identify nine major cell types with 49 subtypes. PXDN expression was primarily expressed in cancer-associated fibroblasts (CAFs), and endothelial cells, with lower expression noted in epithelial cells, cancerous epithelial cells and perivascular-like cells (Figures 3D–3F). The highest expression was observed in CAFs (Figure 3F), matching with the PyMT data (Figure 3C). These findings are also consistent with previous reports for the known role of PXDN in collagen IV stabilization in the basement membrane, in which fibroblasts, epithelial cells, endothelial cells, and cancer epithelial cells have all been shown to express PXDN.^30^^,^^46^^,^^47^^,^^48^^,^^49^^,^^50^^,^^51^^,^^52^^,^^53^^,^^54^^,^^55^^,^^56^

Notably, CAFs are known to be a key stromal cell type involved in ECM production and remodeling in solid tumors. In our datasets, they exhibited the highest PXDN expression among all identified cell types (Figures 3C and 3F). These findings suggest that CAFs are likely a major source of PXDN in the tumor stroma. Given that high stromal staining is associated with poor survival in this cohort (Figure 2F), it raises the possibility that CAF-derived PXDN may contribute to breast cancer prognosis. We, therefore, sought to investigate the functional role of PXDN secreted specifically by CAFs in the development and progression of breast cancer using immunocompetent *in vivo* mouse models.

### Modulating PXDN expression in CAFs alters proliferation and ECM interactions

**Figure 4:**
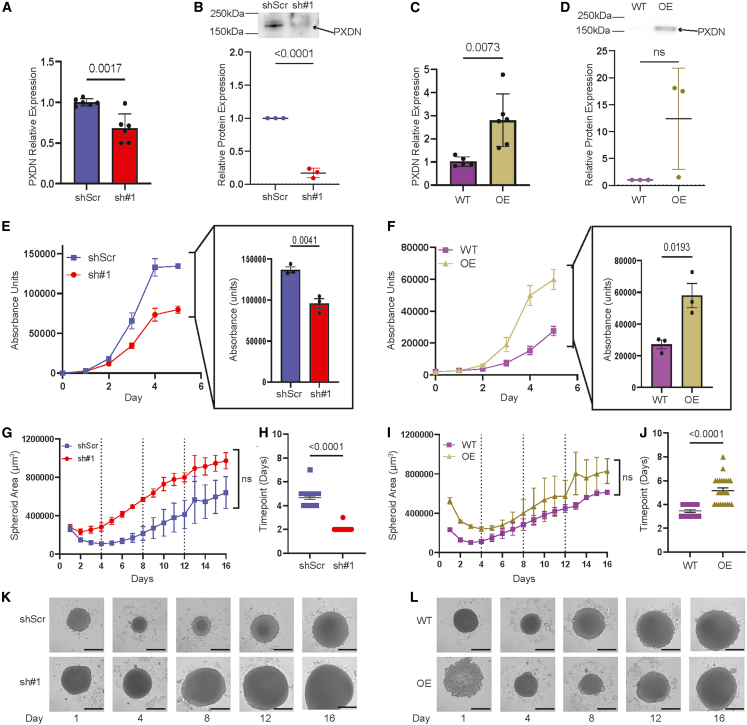
PXDN affects cell proliferation and spheroid formation dynamics in CAFs Differences between shScr CAFs and sh#1 CAFs, or between WT CAFs and OE CAFs, were calculated using Student’s *t* tests and *p* values are shown on each graph. *p* values <0.05 were considered significant. ns = not significant. (A and C) RNA expression of PXDN in shScr and sh#1 (A), or WT and OE (C) CAFs. Error bars show the standard deviation of six independent experiments. (B and D) Representative western blots showing PXDN levels in conditioned media (top) from shScr and sh#1 CAFs (B) or WT and OE CAFs (D), with densitometry quantification of three biological replicate western blots (bottom). Densitometry measurements were normalized to total protein loading (measured by Ponceau S stain) and calculated relative to control (shScr or WT) protein expression. (E and F) 2D proliferation of shScr and sh#1 CAFs (E) or WT and OE CAFs (F) as measured by Alamar blue assay five days of culture (left). Differences between CAF lines were directly compared on day 5 (right). Error bars indicate the standard error of the mean for three biological replicates. (G–L) 3D growth of shScr (blue), sh#1 (red), WT (purple), and OE (gold) CAFs as spheroids over the course of 16 days. (G and I) Spheroid area and (K and L) representative images over the course of 16 days. Scale bars, 400 μm. Dotted lines indicate media changes on days 4, 8, and 12. Error bars indicate the standard error of the mean for three biological replicates. (H and J) The day of spheroid growth at which spheroids reached their smallest size before expanding. Error bars indicate the standard error of 24 spheroids across three biological replicates.

To investigate the functional role of PXDN in breast cancer, CAFs derived from the PyMT model^57^ were genetically modified to either knock down or overexpress PXDN. PXDN knockdown was achieved via lentiviral transduction of shRNA targeting PXDN (sh#1 CAFs), with control CAFs being transduced with a scrambled shRNA construct (shScr CAFs). Knockdown efficiency was confirmed at both the RNA (Figure 4A) and protein (Figure 4B) level. To match this, a complementary model was established in which PXDN was overexpressed in CAFs (OE CAFs) using lipofectamine-mediated transfection of the full length sequence for PXDN in the pcDNA3.1/V5/His-TOPO backbone (a kind gift from Miklós Geiszt^22^), with unmodified parental CAFs (WT CAFs) serving as control. Overexpression was similarly validated at the RNA (Figure 4C) and protein (Figure 4D) level.

To determine if PXDN expression affected the rate of CAF proliferation, an Alamar blue proliferation assay was used. These data show that sh#1 CAFs exhibited a slower proliferation rate compared to shScr CAFs (Figure 4E). In contrast, PXDN OE CAFs had a faster proliferation rate compared to WT CAFs (Figure 4F). To ensure this was not a metabolic effect, differences in proliferation rates between the shScr and sh#1 CAFs, and between WT and OE CAFs, were confirmed by cell counts (Figure S2) and showed the same trends with knockdown reducing proliferation rates, and overexpression increasing proliferation rates in 2D.

Furthermore, sh#1 CAFs showed increased adhesion and spreading dynamics on gelatin-coated surfaces compared to shScr CAFs, while OE CAFs took longer than WT CAFs to completely adhere and spread (Figure S3). PXDN knockdown and overexpression also altered CAF morphology (Figure S4), with sh#1 CAFs exhibiting a more elongated morphology compared to shScr CAFs. Similarly, OE CAFs exhibited a rounder and smaller morphology compared to WT CAFs at 24 h post-seeding. Taken together, these results suggest a role for PXDN in mediating CAF-ECM interactions, thereby affecting cell phenotype.

To assess the impact of PXDN on CAF growth in 3D, CAFs were grown in round bottom plate low adherence cultures in which they coalesce to form spheroids. In this assay, spheroids undergo an initial coalescence phase, where spheroids compact and form, followed by expansion when spheroids increase in area (Figures 4G–4L). shScr CAFs and sh#1 CAFs spheroids formed initially at approximately the same size (Figure 4G). However, shScr CAF spheroids continued to coalesce for longer before expanding in size at around day 5. sh#1 CAF spheroids on the other hand, began to expand in size by day 3 (Figures 4H and 4K). In contrast, the PXDN OE CAFs had the opposite trend when compared to their WT CAF control, whereby OE spheroid cultures took longer to start expanding (around day 5) compared to 3 days for controls (Figure 4J). Interestingly, this differs from the 2D growth rates, where OE CAFs had increased proliferation compared to WT CAFs, and sh#1 CAFs had decreased proliferation compared to shScr CAFs (Figures 4E and 4F). This suggests that factors other than the proliferation rate of CAFs may be contributing to spheroid growth dynamics. It is therefore possible that PXDN overexpression in CAFs may alter cell-cell interactions and/or cell-basement membrane interactions during spheroid formation.

**Figure 5:**
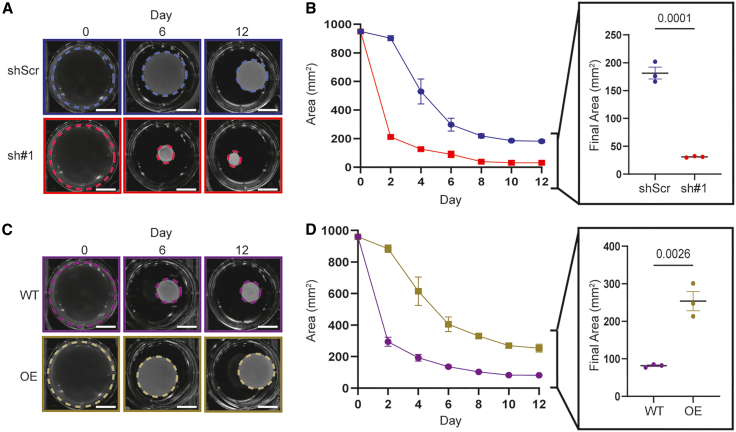
PXDN inhibits the ability of CAFs to remodel collagen matrices (A and C) Representative images of collagen matrices contraction over the course of 12 days when seeded with shScr or sh#1 CAFs (A) or WT or OE CAFs (C). Scale bars, 1 cm. (B and D) Area of matrices during contraction over the course of 12 days (left), with differences in final area of matrices compared at day 12 (right). Error bars indicate the standard error of the mean for three biological replicates. *p* values were calculated using Student’s *t* tests. *p* values <0.05 were considered significant.

In light of these results, we next examined whether PXDN expression alters CAF ability to contract and remodel the ECM. To do this, CAFs were embedded in collagen I rich matrices and matrix contraction, indicative of matrix remodeling, was quantified over 12 days.^58^ PXDN knockdown CAFs exhibited an enhanced ability to remodel the organotypic matrices compared to controls (Figures 5A and 5B), whereas PXDN-overexpressing CAFs showed reduced matrix contraction (Figures 5C and 5D).

Altogether, these data suggest that as PXDN expression in CAFs increases, cell morphology and proliferation rates are altered along with spheroid forming dynamics. Additionally, CAFs with higher levels of PXDN have a reduced ability to remodel and contract collagen matrices. Considering the cell spreading dynamics, spheroid forming dynamics and collagen contractile ability of CAFs, these results indicate that PXDN alters the way CAFs interact with their surrounding environment as well as with one another. The transition from contractile, matrix-organizing CAFs to a more rounded, proliferative phenotype with reduced matrix remodeling ability could disrupt the normal stromal architecture that typically constrains tumor growth, while simultaneously promoting a stromal compartment that supports aggressive cancer cell behavior.

### CAF-derived PXDN activity modulates breast cancer cell behavior

**Figure 6:**
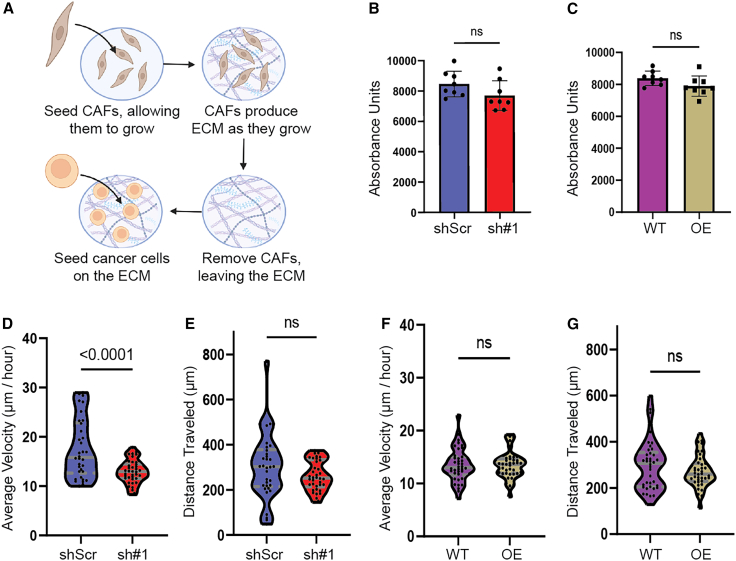
Impact of CAF-produced PXDN on cancer cell proliferation and motility Differences between shScr CAFs and sh#1 CAFs, or between WT CAFs and OE CAFs, were calculated using Student’s *t* tests and *p* values are indicated on each graph. *p* values <0.05 were considered significant. ns = not significant. (A) Schematic of generation of CAF-generated CDMs, onto which cancer cells were seeded. (B and C) Proliferation rates of cancer cells seeded on CDMs produced by sh#1 or shScr CAFs (B), or by WT and OE CAFs (C) as measured by Alamar blue at day 7. Error bars represent the standard deviation of eight replicates. (D and F) Mean velocity of cancer cells when seeded onto CDMs produced by shScr or sh#1 CAFs (D) or by WT or OE CAFs (F). (E and G) Distance traveled by cancer cells when seeded onto CDMs produced by shScr or sh#1 CAFs (E) or by WT or OE CAFs (G). A minimum of ten cells were tracked per biological replicate, across three biological replicates. Error bars indicate the standard deviation.

Given that CAF-derived PXDN influences CAF proliferation, phenotype and ECM remodeling, we next investigated whether PXDN-dependent changes to the ECM would affect cancer cell proliferation and motility. To model this, cell-derived matrices (CDMs)^59^^,^^60^ were generated by CAFs with either PXDN knockdown or overexpression. After matrix deposition, the CAFs were removed, and PyMT cancer cells were seeded onto the remaining CAF-derived matrix (Figure 6A).

Interestingly, Alamar blue assays showed no significant differences in proliferation rates of cancer cells seeded on CDMs derived from sh#1, shScr, OE, or WT CAFs (Figures 6B and 6C). Similarly, live-cell imaging confirmed no significant differences in cell-cycle duration (measured from one division to the next) (Figure S5). In contrast, cancer cell motility was influenced by the composition of the CDM. Cells seeded onto CDMs derived from PXDN knockdown CAFs (sh#1) exhibited decreased motility, traveling at lower velocities (p = <0.001) (Figure 6D) compared to those on control (shScr) matrices and trending toward traveling shorter distances (Figure 6E). Conversely, cancer cells on OE CAF-derived CDMs showed no statistically significant differences in motility in this assay (Figures 6F and 6G).

These findings show that although PXDN-mediated ECM remodeling by CAFs does not appear to impact cancer cell proliferation, it does alter cancer cell motility. This suggests a role for PXDN in mediating cancer cell-ECM interactions that influence cell motility.

### *In vivo* effects of PXDN modulation in CAFs in an *in vivo* orthotopic model of breast cancer

Following the *in vitro* studies demonstrating that CAF-derived PXDN modulates CAF and cancer cell phenotype and behavior, we next investigated the *in vivo* effects of PXDN knockdown or overexpression in CAFs on tumor growth in an immunocompetent, syngeneic co-implantation mouse model. To do this, an orthotopic model of breast cancer was established by co-implantation of PyMT-derived cancer cells, together with either sh#1, shScr, OE, or WT CAFs (1:3 ratio) into the 4^th^ mammary fatpad of FVB mice. This co-implantation approach was employed since CAFs are known to alter tumor growth dynamics (Figure S6E), so this model allows for primary tumor formation while enabling the specific study of stromal CAF-derived PXDN on tumor establishment and progression. Following implantation, tumors were allowed to grow until they reached an ethical endpoint size of 1 cm × 1 cm (equivalent to 520 mm^3^) as a time-to-matched endpoint study.

**Figure 7:**
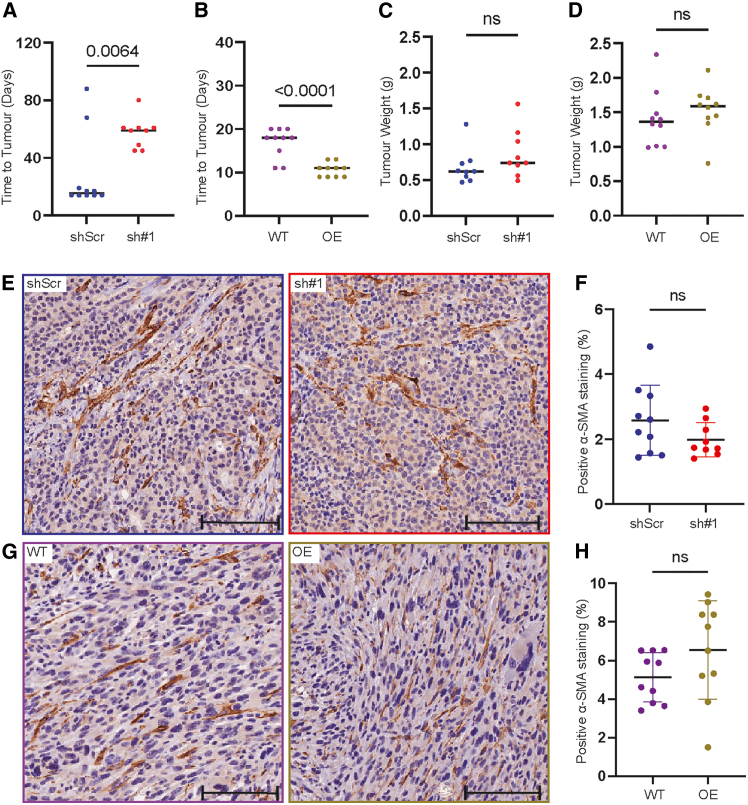
Effect of CAF PXDN expression on breast tumor progression *in vivo* Differences between shScr CAFs and sh#1 CAFs, or between WT CAFs and OE CAFs, were calculated using Student’s *t* tests and *p* values are indicated on each graph. *p* values <0.05 were considered significant; ns = not significant. (A and B) Latency of tumor formation from the time cells were implanted into mice along with shScr or sh#1 CAFs (A) or with WT or OE CAFs (B) until tumors reached 50 mm^3^ in size. (C and D) Final weights of tumors upon collection from mice implanted with cancer cells and shScr or sh#1 CAFs (C) or with WT or OE CAFs (D). (E) Representative images of α-SMA staining in tumors from shScr or sh#1 CAF tumors. Scale bars, 100 μm. (F) Quantification of α-SMA staining in shScr and sh#1 CAF tumors. Error bars indicate the standard deviation. (G) Representative images of α-SMA staining in tumors from WT or OE CAF tumors. Scale bars, 100 μm. (H) Quantification of α-SMA staining in WT and OE CAF tumors. Error bars indicate the standard deviation.

Median survival, measured from the time of cell implantation until the endpoint tumor size (maximum possible tumor size based on animal ethics), did not significantly differ between mice implanted with sh#1 versus shScr CAFs (Figure S6A), nor between those with OE versus WT CAFs (Figure S6B). Strikingly, the latency time (determined as time from implant to palpable tumor formation; defined as > 50 mm^3^) for tumors containing sh#1 CAFs was significantly longer compared to tumors co-implanted with shScr CAFs (*p* = 0.004) (Figures 7A and S6C), suggesting that reduced stromal PXDN slows early tumor establishment. In contrast, the latency period was shorter with tumors forming more rapidly in mice implanted with OE CAFs compared to WT CAFs (*p* = <0.001) (Figures 7B and S6D). Since tumor volume determined the study endpoint in this experiment, we confirmed that final tumor weights were comparable between sh#1 and shScr groups (Figure 7C), as well as between OE and WT groups (Figure 7D), validating that tumor size estimates were consistent. Our data suggest that high expression of PXDN during earliest stages of tumor progression, as seen in the proteomics data (Figure 1B), may correspond to a period in which PXDN is promoting tumor initiation, although this effect appears to diminish as tumors progress.

To confirm that these observed effects were not due to differences in CAF presence within the tumor, tissue sections were stained and scored for the CAF marker alpha smooth muscle actin (α-SMA) (Figures 7E–7H). These data confirmed no statistically significant differences in total α-SMA positivity across tumors from different groups; however, we acknowledge that these CAFs are likely a mix of both the co-implanted CAFs along with locally recruited and activated mammary fibroblasts, since α-SMA positive staining is also observed in tumors from mice implanted with cancer cells only (Figure S7A). Given the presence of recruited host CAFs in tumors, we next sought to confirm the continued presence of our implanted CAFs in tumors which express a GFP tag within the short hairpin construct. GFP was present in all sh#1 and shScr CAF co-implanted tumors in the knockdown study, showing that implanted CAFs remain in the tumors at endpoint (Figure S7B).

Finally, lung tissues collected at endpoint were scored for metastases but showed no significant differences in metastatic burden between groups, although metastasis in this model is usually low (Figure S8) in the absence of tumor resection.

### Pharmacological inhibition of PXDN in an *in vivo* orthotopic model of breast cancer

**Figure 8:**
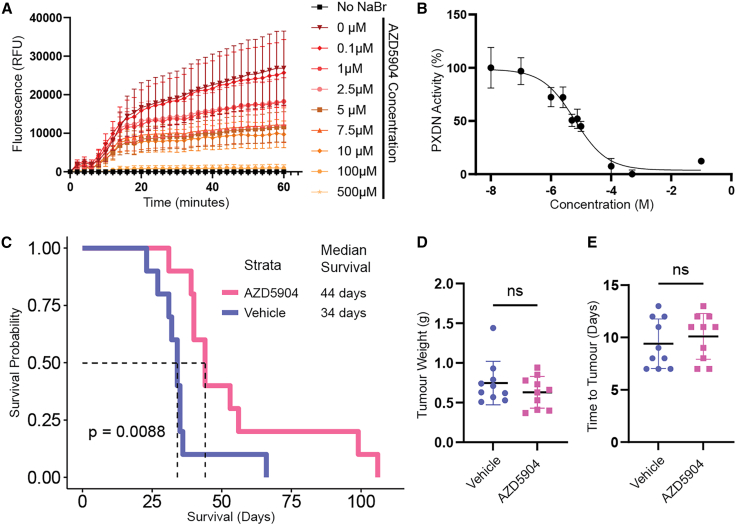
Effect of PXDN inhibition on breast tumor progression using AZD5904 (A) Inhibition of PXDN by a range of concentrations of AZD5904 measured on live cells using a modified Amplex red activity assay. Resorufin fluorescence was monitored continuously for 1 h. Error bars indicate standard error of the mean across three biological replicates, each consisting of eight technical replicates. (B) Final resorufin fluorescence readings at 60 min were used to determine to IC50 inhibitory concentration of AZD5904. (C) Kaplan-Meier curve of mouse survival measured from the start of treatment with AZD5904 or vehicle (when tumors reached a detectable size of 50 mm^3^) until tumors reached a maximum ethical size of 1 cm by 1 cm and mice were sacrificed. Survival curves were significantly different, with a log-rank *p* value of 0.0088. (D) Final weights of tumors upon collection from mice in the AZD5904 *in vivo* study. Error bars indicate the standard deviation. Student’s *t* test showed no statistical significance between vehicle and AZD5904 treatment groups. (E) Time taken for tumors to reach the detectable size of 50 mm^3^ at which point treatment started. Error bars indicate the standard deviation. Student’s *t* test showed no statistical significance between vehicle and AZD5904 treatment groups.

To further assess the clinical relevance of targeting PXDN, and to overcome any effects that may be as a result of locally recruited mammary fibroblasts, we sought to test potential pharmacological PXDN inhibition of tumor growth. There are currently no PXDN-specific inhibitors available; however, AZD5904 is a small molecule peroxidase inhibitor originally developed to block the activity of myeloperoxidase. It has previously been shown to be well tolerated in humans and cleared phase I clinical trials.^61^ Both PXDN and myeloperoxidase rely on a central heme group within their active site, which is essential for peroxidase activity. As such, we first evaluated AZD5904 for its ability to inhibit PXDN activity using a live-cell Amplex Red assay adapted to detect PXDN activity.^62^ We found that AZD5904 was able to inhibit PXDN activity in CAFs in a dose-dependent manner, with an IC_50_ of 6.1 μM (95% CI [2.4 μM, 13.8 μM]) *in vitro* (Figures 8A and 8B).

The therapeutic potential of AZD5904 was then tested *in vivo* using the same orthotopic co-implantation model (PyMT cancer cells + shScr CAFs). The CAF and epithelial cancer cell lines are derived from the genetically engineered PyMT mouse model, which show high expression of PXDN in CAFs and low expression in cancer cells in the single cell RNAseq data (Figures 3A–3C). The orthotopic co-implantation mouse model therefore recapitulates the high stromal and low epithelial PXDN expression group shown to have the worst overall survival in our TMA cohort (Figure 2G). Once tumors reached a detectable size (>50 mm^3^), mice were treated twice daily with 75 mg/kg AZD5904 via oral gavage.

Complete blood count analysis was performed on blood collected at endpoint to determine whether AZD5904 treatment elicited any systemic off-target effects. No negative effects on red blood cell parameters were observed during AZD5904 treatment compared to vehicle (Figures S9A–S9C), confirming that AZD5904 did not induce bone marrow toxicity. Furthermore, no differences were observed in white blood cell populations, further demonstrating that AZD5904 did not alter leukopoiesis or circulating immune cell populations (Figures S9D–S9F). Additionally, similar to the genetic modulation experiments, there was no significant difference in metastatic burden in AZD5904-treated vs. control mice (Figure S10).

However, mice treated with AZD5904 showed improved median and overall survival compared to vehicle-treated controls (*p* = 0.0088), measured from treatment initiation to endpoint tumor size (Figure 8C). Final tumor weights were similar between groups (Figure 8D), confirming consistent volumetric endpoint measurements. There was no difference in the time taken for tumors to develop prior to the start of AZD5904 treatment (Figure 8E), indicating that differences in survival were a result of AZD5904 treatment. As time to study endpoint was determined by tumor volume, these data confirm that AZD5904 shows promise in slowing tumor growth and improving outcome in *in vivo* breast cancer models.

## Discussion

In this study, we demonstrate that PXDN plays a stage-dependent role in breast cancer progression, characterized with peak expression during early tumor development and exerting compartment-specific effects on patient outcome. Stromal PXDN, predominantly produced by CAFs, appears to promote early tumor establishment through matrix remodeling mechanisms that enhance cancer cell motility, and pharmacological inhibition of all PXDN improves survival in preclinical models.

Our temporal proteomic analysis revealed that PXDN expression follows a distinct pattern during breast cancer progression, with peak expression occurring during early-to-mid stage disease in mouse models. This finding was corroborated in human patients, where high PXDN expression was significantly associated with poor outcomes specifically in stage II breast cancer patients. The stage-dependent nature of PXDN’s prognostic value suggests that its role may be most critical during the establishment of invasion-permissive niches that support tumor cell invasion. Given that PXDN is not associated with patient survival in later stages of breast cancer, it may be that PXDN only exerts its role early in disease progression while other factors become the dominant drivers of disease once tumors are established. However, a role for PXDN in long-term metastatic outgrowth cannot be formally excluded based on data from our experiments and further work should be conducted to establish the stage-dependent effects of PXDN, for example against occult and established metastatic disease. This would be particularly important when testing future therapies targeting PXDN that may be more efficacious in different disease settings.

Compartment-specific analysis of PXDN expression revealed opposing associations with patient survival depending on cellular localization. High stromal PXDN correlated with poor outcomes, while high epithelial PXDN was associated with better survival. This compartmentalization likely explains why previous pan-cancer analyses failed to identify PXDN as prognostically significant in breast cancer,^26^ as bulk expression measurements would average these opposing signals. Our single-cell analysis confirmed that CAFs are likely the primary stromal source of PXDN in tumors, positioning them as key orchestrators of PXDN-mediated tumor microenvironment remodeling.

Our data demonstrate that PXDN modulates fundamental CAF behaviors associated with altered ECM which influence disease progression including proliferation, morphology, cell-ECM interactions, and most notably, matrix contractility. The knockdown of PXDN in CAFs leading to reduced motility of cancer cells seeded onto CAF-derived extracellular matrices suggests that PXDN acts extracellularly upon cancer cells. Certainly, the selective effect of PXDN on motility rather than proliferation aligns with PXDN’s role in early disease progression, where enhanced invasive capacity may be more critical than proliferative advantage in affecting overall patient survival. This effect of PXDN may be through its canonical function in crosslinking of collagen IV.^22^^,^^23^^,^^24^ However, PXDN has also been shown to bind to laminin in the ECM,^63^ and has been reported to have other putative intracellular functions,^46^ so the exact mechanisms by which PXDN moderates cell behavior remain to be resolved.

A number of other enzymes are known to remodel the ECM, including the lysyl oxidase family of enzymes involved in fibrillar collagen crosslinking^64^^,^^65^ and matrix metalloproteinases which degrade these collagens.^38^^,^^66^^,^^67^ It will be an important area of future work in this field to investigate whether PXDN expression and activity impacts CAF secretion of these other matrix modifying enzymes and how this interplay may affect ECM deposition, remodeling and cross-linking in cancer.

Knockdown of PXDN had a larger effect on CAF 3D growth, cancer cell motility and *in vivo* tumor formation than overexpression of PXDN. It is possible that the secreted levels of PXDN in WT CAFs were sufficient to support normal physiological functions of PXDN, and so overexpression did not lead to statistically significant alterations in cell behavior, particularly in the case of cancer cell motility. Again, it will be important to elucidate the exact mechanism by which PXDN exerts its changes on the ECM and on surrounding cells to determine why overexpression of PXDN is less impactful than knockdown of PXDN in this model.

Our preclinical therapeutic studies with AZD5904 demonstrated the translational potential of targeting extracellular peroxidases in breast cancer. The survival benefit observed with AZD5904 treatment exceeded that seen with genetic CAF-specific PXDN knockdown, likely reflecting the compound’s ability to inhibit PXDN from all cellular sources simultaneously. With that in mind, it will be important for future work to further investigate the role of epithelial-derived PXDN in breast tumor progression as this may differ from its stromal role. In particular, previous studies have identified that knockdown of PXDN alterations in ovarian, prostate and gastric cancer and melanoma cell lines lead to decreases in cell migration, invasion and anchorage-independent growth.^46^^,^^47^^,^^48^^,^^68^^,^^69^ Additionally, AZD5904’s capacity to inhibit myeloperoxidase, another known tumor-promoting peroxidase,^70^^,^^71^^,^^72^ may contribute synergistically to its therapeutic efficacy.

The excellent tolerability profile observed in our studies, combined with AZD5904’s successful completion of phase I clinical trials, supports its potential for future clinical translation, and presents a compelling case for clinical evaluation in breast cancer patients. Given the stage-dependent effects observed, early-stage patients with high stromal PXDN expression may represent an ideal population for initial clinical trials.

Looking forward, our work reinforces extracellular peroxidases as a potential matrix-targeting therapeutic opportunity, as well as incorporating stromal biomarkers like compartmentalized PXDN expression into treatment decisions to support patient stratification.

### Limitations of the study

Some limitations warrant consideration. The broad-spectrum nature of AZD5904 means that the observed therapeutic effects likely result from inhibition of multiple peroxidases beyond PXDN alone. While this may enhance therapeutic efficacy, it complicates mechanistic interpretation and highlights the need for more selective PXDN inhibitors in future studies. Additionally, myeloperoxidase, an enzyme primarily expressed by neutrophils, macrophages and monocytes, has been shown in previous studies to promote tumor growth through multiple mechanisms involving both tumor cells and the tumor microenvironment,^70^^,^^71^^,^^73^ which could also influence outcomes. Furthermore, since AZD5904 showed a beneficial effect over and above that seen in the genetically manipulated CAF experiments, it suggests that PXDN from other sources in addition to that from CAFs may also be playing a role in determining outcomes.

The high expression of PXDN by CAF populations in our single cell data led us to focus our attention on CAF-derived PXDN in this model, and how this alters the ECM and in turn affects cancer cell behavior. However, we did not explore the impact of PXDN on immune cell function and infiltration within the tumor microenvironment. We were also unable to investigate whether PXDN secreted locally by the tumor is acting systemically at secondary organs, or in the generation or pre-metastatic niches. These remain important areas of future study. We further identified that PXDN protein expression was associated with tumor grade; however, we were unable to further explore the relationship between CAF expression of PXDN and grade in our orthotopic models since the PyMT model develops murine tumors that are all poorly differentiated.^34^ Further work will be needed to better understand this association.

The precise molecular mechanisms underlying PXDN’s effects on CAF phenotype and matrix properties remain to be fully elucidated. Future studies employing advanced matrix characterization techniques, including atomic force microscopy and cryo-electron tomography, will be essential to understand how PXDN-mediated crosslinking affects matrix mechanics and architecture at the nanoscale level.

## Resource availability

### Lead contact

Requests for further information and resources should be directed to and will be fulfilled by the lead contact, Thomas Cox (t.cox@garvan.org.au).

### Materials availability

The PXDN manipulated CAF cell lines are available upon reasonable request with a material transfer agreement.

### Data and code availability

This study analyses existing, publicly available data. Proteomics data from the PyMT mouse model is accessible at MassIVE (MSV000089167) and is also available in ProteomeXchange (accession code PXD032876).^31^ TCGA RNA-seq data from patients with invasive breast cancer is accessible at the International Cancer Genome Consortium (ICGC) (June, 2021).^74^ All original code is available in this study’s supplemental information. Any additional information required to reanalyze the data reported in this study is available from the lead contact upon request.

## Acknowledgments

The authors acknowledge the facilities as well as scientific and technical assistance at the Australian Bioresources (ABR), the Garvan ACRF INCITe Imaging Centre and the Garvan Histology Core Facility. This work was supported by the 10.13039/501100000925NHMRC (Q.E.T., E.C.F., J.L.C., R.E., A.L.P., and T.R.C.), 10.13039/501100001102Cancer Council NSW (A.L.P. and T.R.C.), 10.13039/501100001171Cancer Institute NSW (A.L.P.), and Australian Government Research Training Program Scholarships (K.W. and E.T.Y.M.). This research was supported by 10.13039/501100022794Love Your Sister in association with the 10.13039/501100001026National Breast Cancer Foundation, Australia.

## Author contributions

Conceptualization and methodology, K.W., A.L.P., and T.R.C.; investigation, K.W., E.T.Y.M., Q.E.T., E.C.F., J.L.C., R.E., A.Z., A.M.D.S., M.T., and S.O.’T.; resources, D.G.-O. and S.O.’T.; funding acquisition, V.P. and T.R.C.; supervision, A.L.P., V.P., and T.R.C. writing and editing, K.W., A.L.P., V.P., and T.R.C.

## Declaration of interests

The authors declare no competing interests.

## STAR★Methods

### Key resources table

**Table:** 

REAGENT or RESOURCE	SOURCE	IDENTIFIER
**Antibodies**		

PXDN (VPO1)	Sigma-Aldrich	ABS1675
GFP	ThermoFisher Scientific	A11122
Vinculin	Sigma-Aldrich	V9131
α-SMA	Abcam	AB5694
Anti-rabbit HRP linked	New England Biolabs	7074S
Anti-mouse HRP linked	New England Biolabs	7076

**Bacterial and virus strains**		

DH5α E.coli	ThermoFisher Scientific	Cat#18265017

**Biological samples**		

CREA tumor microarray	Millar et al.^75^	NA

**Chemicals, peptides, and recombinant proteins**		

Lipofectamine 2000	ThermoFisher Scientific	Cat#FNP9404193
Alamar Blue	Invitrogen	Cat#DAL1100
Amplex red	ThermoFisher Scientific	Cat#A12222
AZD5904	Sapphire Bioscience	Cat#BS-82781C

**Experimental models: Cell lines**		

HEK293T	ATTC	CRL-3216
PyMT cancer cells	Floerchinger et al.^76^	NA
PyMT CAFs	Calvo et al.^57^	NA

**Experimental models: Organisms/strains**		

Mouse: FVB/JAusb	Australian Bio Resources	NA

**Recombinant DNA**		

recombinant PXDN cloned into a pcDNA3.1/V5/His-TOPO backbone	Lázár et al.^22^	NA
Lentiviral PXDN shRNA knockdown constructs	OriGene	TL513178V

**Software and algorithms**		

ImageJ	National Institute of Health	https://imagej.nih.gov/ij/
R	Comprehensive R Archive Network	https://cran.r-project.org/bin/windows/base/
GraphPad Prism	GraphPad	https://www.graphpad.com/scientific-software/prism/
Perseus	MaxQuant	https://maxquant.net/perseus/
QuPath	QuPath	https://qupath.github.io/

### Experimental model and study participant details

#### Cell lines

Cell lines (CAFs and cancer cells) were derived from the PyMT mouse model of breast cancer. PyMT CAFs were obtained from Fernando Calvo^57^ and cultured in DMEM +10% FBS +1% pen/strep +1% Insulin, Selenium, Transferrin (ITS). PyMT20065 cancer cells were derived with the support of Dr. Karen Blyth at the CRUK Beatson Institute in Glasgow and have been previously published.^31^^,^^76^ Cells were cultured in DMEM (Gibco) +10% FBS +1% pen/strep +5 μg/mL insulin +10 ng/mL epidermal growth factor +10 ng/mL Cholera Toxin A. HEK293T cells were cultured in DMEM +10% FBS +1% pen/strep. All cells were cultured in a humidified incubator at 37°C, 21% O_2_ and supplemented with 5% CO_2_. All cell lines were of female origin and were routinely confirmed negative for mycoplasma.

#### Mouse models

Mice used in this study were FVB/n strain female mice (aged 11 weeks) bred at the Australian BioResources Pty Ltd (ABR) facility in Moss Vale, NSW. Animals were randomly assigned into experimental groups. All animal experimentation was approved by the Garvan and St Vincent’s Precinct Animal Ethics Committee (ARA 19_08, ARA 22_04) and followed recommendations in the Australian Code of Practice for the Care and Use of Animals for Scientific Purposes by the National Health and Medical Research Council.

### Method details

#### Mass spectrometry data analysis

Mass spectrometry files containing protein abundance data from decellularised PyMT mammary tumors at early (8–10 weeks of age), mid (11–13 weeks of age) and late (14–16 weeks of age) stages of disease progression, along with age-matches samples from healthy mouse fat pads, were obtained from MassIVE (MSV000089167) and are also available in ProteomeXchange (accession code PXD032876).^31^ Data was imported into the Perseus software package (version 1.6.15.0) for data filtering. Proteins that were present in less than 50% of technical replicates across all of the biological groups (healthy or tumor samples at early, mid or late stages of disease), were filtered out of the dataset. The dataset was further filtered to retain only ECM proteins by comparison to the known *Mus musculus* ECM protein listed in the Matrisome Project (rev 2014) from the Naba Lab.^35^^,^^77^ Data was then Log2 transformed and individual samples with more than 55% missing values for all proteins were removed from the dataset.

Data was imputed in a group wise manner (based on the biological groupings of tumor or heathy and stage of disease) by replacing missing values with the lowest recorded value for that group in cases where at least 60% of values for a given protein within a biological group were present. If less than 60% of values in a group were present, no imputation was performed. In cases where all values for a given protein in one group were missing, missing values were replaced with 0.^78^

Data was then imported back into the Perseus software package (version 1.6.15.0). The variance between samples was visualised by generating PCA plots in Perseus using filtering data to only contain proteins with no missing values before PCA with the Benjamini-Hochberg cut-off method and an FDR of 0.05. Significantly differentially expressed (*p* < 0.05) proteins between groups within each dataset were identified by MANOVA with an FDR of 0.05. Grouping of samples for MANOVA was determined based on clustering of samples in PCA plots and were as follows; all healthy samples, early and mid-stage tumor samples, and late-stage tumor samples. For heatmap generation, *Z* score normalisation was applied to data before generation of heat maps with protein clustering according to Euclidean distance.

#### Survival analysis in TCGA cohorts

Publicly available microarray and corresponding anonymised clinical data from 1,082 patient samples with invasive breast cancer from The Cancer Genome Atlas (TCGA) cohort was obtained from the International Cancer Genome Consortium (ICGC) (June, 2021).^74^ Survival analysis was performed using R. Patients were stratified into high and low expressing groups based on the median gene expression value and their association with overall survival was visualised using the Survminer (version 0.5.0) and ggplot2 (version 3.5.2) packages to generate Kaplan Meier survival curves. For overall survival, patients that died of causes other than cancer were censored. *p*-values were calculated using the log rank test using the Survminer package and were considered significant if *p* < 0.05. Overall survival was also analyzed using the online Kaplan-Meier Plotter (https://kmplot.com/analysis/) using mRNA gene chip expression data in breast cancer. PXDN (Affy ID 212012_at) was stratified by median expression with all follow up thresholds and all subtypes included. No restrictions or exclusion criteria were applied. A total of 1,879 patients were included in the survival analysis.

#### TMA analysis

Paraffin embedded tumor tissue microarrays (TMA) from the CREA breast cancer cohort^75^ were cut into 4 μm sections and baked onto Superfrost plus slides at 60°C (ThermoFisher Scientific, 491PLUS4). Sections were then deparaffinised in Bond Dewax (AR9222) + 100% ethanol. Heat induced epitope retrieval was performed at pH 9 (Leica, AR9640) for 30 min at 100°C. PXDN (1:750, Merck, ABS1675) staining was performed using the Bond RX Austostainer (Leica) using the Bond Polymer Refine Detection kit (Leica, DS9800) with an incubation of 30 min. Slides were counterstained with Haematoxylin (Australian Biostain Haematoxyli Harris non-toxic, acidified) and cover slipped. Slides were digitised using the Nanozoomer S210 (SDR Scientific).

Tumor cores were scored according to the percentage coverage of stain and intensity of stain. Intensity was scored on a scale of 0–3 for stromal and epithelial compartments separately, where 0 was no staining and 3 was the highest intensity staining. All tumors had some level of epithelial staining. Percentage of coverage within each compartment was scored manually so that <10% coverage = 0, 11–50% Coverage = 1, 51–90% coverage = 2 and >90% coverage = 3. Coverage scores were multiplied by intensity scores to obtain a final Allred-score for each compartment. A total stroma score of 0–3 (that <10% coverage = 0, 11–50% Coverage = 1, 51–90% coverage = 2 and >90% coverage = 3) was also noted, which reflected the proportion of stroma compared to epithelia within the core. Stromal and epithelial Allred scores were then stratified into PXDN high and low groups. In contrast to RNA-seq expression values, Allred scores are not continuous variables, and we sought to use stratification thresholds that reflected the distribution of this data. A majority (69%) of epithelial scores were 9, the highest possible score, with a substantial left tail. Therefore, we performed an approximate tertile stratification where all scores of 9 were considered high (69%) and scores below 9 (31%) were considered low epithelial expression. Similarly, stromal Allred scores of 6 or 9 were considered high and stromal scores below 6 were considered to be low. Both stromal and epithelial PXDN Allred scores were calculated within the same patient in all cases where patients had both stromal and epithelial tumor compartments present in each core.

Chi-squared tests were used to test for differences between the spread of high and low PXDN expression tumors within different treatment and molecular subtype groups. Since grade had small groups, a Fishers exact test was utilised.

Clinical information (survival, age and treatment) was provided for the majority of patients in the cohort (Table 2). Grade and molecular subtype (determined by Ki67, ER, PR and HER2 status) had been previously calculated by senior pathologist Sandra O’Toole. Survival analysis was performed in R (version 4.0.4, RStudio version 1.4.1103). Patients with tumor types other than IDC or missing survival information were removed prior to survival analysis. The Survminer (version 0.5.0) and ggplot2 (version 3.5.2) packages were used to generate Kaplan Meier survival curves stratifying for PXDN expression in the stromal and epithelial compartments of tumors and molecular subtype. Cores with no stromal tissue, or with no epithelial tissue, were excluded from survival analysis. *p*-values were calculated using the log rank test and were considered significant if *p* < 0.05. Univariate and multivariate analyses was performed using the Survival package (version 3.3.1).

#### Single cell analysis

PyMT mouse model single cell data was obtained from the Valdes-Mora et al. dataset (GSE158677).^43^ As performed previously, a total of 11,490 cells were included from five tumors from the MMTV-PyMT genotype and subsequent analysis was performed using the Seurat (version 3.2) package.^31^ QC thresholds were set at cell calling of <5% mitochondrial to nuclear gene content and <8000 molecules/cell. Downstream analysis was performed as previously described.^79^ Briefly, a K-nearest neighbor (KNN) graph was built using 30 principal components “FindNeighbors” and default “FindCluster” parameters. Data visualisation was performed using non-linear dimensional reduction UMAP.

Expression levels of human PXDN in different cell types was investigated using the single cell data generated by Wu et al. from a cohort of 26 primary tumors from patients with invasive breast cancer (11 luminal breast cancers, 5 HER2+ breast cancers and 10 triple negative breast cancers).^45^ A total of 130,246 single cells were annotated in this data. Data was accessed using the Broad Institute Single Cell Portal,^44^ and Uniform Manifold Approximation and Projection (UMAP) plots and volcano plots generated using the web interface.

#### Knockdown of PXDN in PyMT CAFs

Lentiviral PXDN shRNA knockdown constructs and a Scrambled control construct containing GFP sequences were obtained from OriGene (TL513178V). Constructs were introduced into competent DH5α *E*.*coli* cells by heat shock transformation, scaled up, and DNA subsequently purified using a MaxiPrep DNA purification kit (ThermoFisher Scientific). HEK293T cells were seeded at a density of 3 x 10^5^ cells per well in a 6-well plate in DMEM +1% FBS without antibiotics and incubated for 24 h. Lipofectamine 2000 (Thermofisher) was diluted in Opti-MEM (Gibco) and incubated at room temperature for 30 min. The lentiviral shRNA PXDN knockdown constructs were diluted to 48.5 ng/μL in Opti-MEM. 3^rd^ generation lentiviral packaging mix^80^ was created by mixing 22.5 ng/μL pMDLg/pRRE, 32.5 ng/μL pRSV-Rev and 14 ng/μL pMD.G in Opti-MEM. Packaging mix was added to the lentiviral construct DNA, and the lipofectamine mix, and incubated at room temperature for 45 min to form the transfection mix. HEK293T cells were incubated with this transfection overnight. Media was changed to fresh medium and incubated for a further 24 h before viral particles were collected and filtered through a 0.45 μm filter. Fresh medium was again added to transfected HEK293T cells incubated for 24 h before additional viral particles were again collected, filtered through a 0.45 μm filter.

For lentiviral infection, PyMT CAFs were seeded at 4 x 10^4^ cells per well in a 6-well plate in 1 mL per well DMEM +1% FBS +1% ITS without antibiotics and incubated overnight. The next day, freshly collected and filtered virus was mixed with polybrene (8 μg/mL). Media was removed and 1 mL viral mixture was then added per well. After a further 24 h, media was replaced with fresh media. 48 h later, 18 μg/mL puromycin was added to the media. After culture under selection for 4 passages, GFP positive populations were sorted for by FACs. Cells within the bottom 40% GFP signal or top 10% GFP signal were discarded, and the remaining cells collected. Following sorting, cells were maintained in media containing 18 μg/mL puromycin for an additional two weeks prior to use in further studies. Maintenance of GFP expression was confirmed periodically by microscopy during maintenance and prior to *in vivo* experiments.

#### Overexpression of PXDN in PyMT CAFs

The open reading frame of recombinant PXDN cloned into a pcDNA3.1/V5/His-TOPO backbone was kindly donated by Miklos Geiszt.^22^ Constructs were introduced into competent DH5α *Escherichia coli* cells by heat shock transformation, scaled up, and DNA subsequently purified using a MaxiPrep DNA purification kit (ThermoFisher Scientific). PyMT CAFs were seeded into a 6-well plate at a density of 3.5 x 10^5^ cells per well in DMEM +1% FBS without antibiotics. 4 μg of recombinant PXDN DNA was diluted in Opti-MEM Reduced Serum Medium (Gibco) and mixed by gently inverting the tube twice. For a control mock transfection, 4 μL of H_2_O was diluted in Opti-MEM. Lipofectamine 2000 diluted Opti-MEM and mixtures were then combined before a further 20-min incubation at room temperature. 250 μL of combined lipofectamine and DNA mix was added to cells in 2 mL of DMEM +1% FBS without antibiotics. Cells were incubated at 37°C for 4–6 h before media was removed and replaced with standard media without selective antibiotics. 24 h after transfection, media was exchanged to medium containing selective antibiotics (DMEM +10% FBS +1% pen/strep +1,500 μg/mL geneticin) and cells were continuously grown under selection medium for 6 weeks prior to use in further experimental work. Control WT CAFs with mock transfection did not undergo antibiotic selection.

#### Western blots

PyMT CAFs (PXDN knockdown, overexpression and controls) were seeded at a density of 1.5 x 10^6^ cells per T150 tissue culture flask for 24 h. Cells were then washed quickly 2x with PBS before 20 mL of Phenol red-free, FBS free DMEM was added to cells, which were then grown for a further 96 h. Conditioned media was collected from the flask, centrifuged at 300xg for 5 min to pellet cellular debris, and supernatant filtered through a 0.45 μm filter. Conditioned media was then concentrated 100x in Amicon Ultra 100 kDa spin columns (Merck) and buffer exchanged with two changes of PBS by centrifugation at 5,000xg at 4 °C. To generate tumor lysates, approximately 5 mg of tumor tissue was lysed using a pulse ultrasonicator (QSonica) at 80 kHz in lysis buffer (0.5M HEPES, 0.1% Triton X-100, 0.5% NaDoc, 1% SDS, 0.02% NaF, 5 mM EDTA) at intervals of 30 s (with cooling on ice in-between sonication) until no solid tissue remained.

Total protein concentrations were measured using the Pierce BCA Protein Assay (ThermoFisher Scientific) as per the manufacturer’s protocol. Samples were diluted to 1 μg/μL in lysis buffer (0.5M HEPES, 0.1% Triton X-100, 0.5% NaDoc, 1% SDS, 0.02% NaF, 5 mM EDTA) before being mixed with NuPAGE LDS sample buffer with 10% β-Mercaptoethanol (Invitrogen). Samples were then heated to 75°C for 10 min before an equal 15 μg total protein was loaded per well and run on a NuPAGE 4–12% Bis-Tris gel (Invitrogen) in NuPAGE MOPS SDS running buffer (Invitrogen) at 100V for 2 h 15 min. Protein transfer onto an Immobilon-P PVDF transfer membrane (Merck Millipore Ltd.) was performed for 75 min at 30V in NuPAGE Transfer Buffer (Invitrogen) + 10% methanol. Membranes were equilibrated in water for 5 min and then incubated with 0.1% Ponceau S for 5 min with gentle rocking. Membranes were washed briefly with water and imaged on a FusionFx7 digital imager. Membranes were then washed and destained in Tris-buffered saline +0.1% Tween 20 for 5 min before antibody blotting.

Membranes were blocked in 10% skim milk powder in Tris-buffered saline +0.1% Tween 20 for 60 min at room temperature before incubation with either the PXDN (1:750 dilution, ABS1675, Merck) or GFP (1:1000 dilution, A11122, ThermoFisher Scientific) primary antibodies prepared in 5% BSA in TBST. Membranes were incubated in primary antibody solutions overnight at 4°C with gentle rocking. Membranes were then washed 3x with TBST for 15 min each wash, followed by a 1-h incubation at room temperature with anti-rabbit HRP secondary antibody (1:5,000, 7074S, New England Biolabs) diluted in 5% skim milk powder in TBST. Membranes were then washed 3 × 15 min each with TBST and imaged using ECL Ultra (PerkinElmer) chemiluminescent detection substrate on a FusionFx7 digital imager using automated exposure times. For GFP blots, post imaging, the membranes were washed 5x with TBST before incubation at 4 overnight with Vinculin (housekeeping) antibody (1:1,000, V9131) made up in 5% BSA in TBST. The membrane was then washed as above, incubated for 1 h at room temperature with anti-mouse HRP secondary antibody (1:10,000, #7076, New England Biolabs), before being washed once more and imaged as above. Densitometry was performed on western blot images using mean gray value measurements in ImageJ. Densitometry measurements were normalised to total vinculin and calculated relative to control (shScr or WT) protein expression.

#### RT-PCR

PyMT CAFs (1 x 10^6^) were pelleted at 300xg for 5 min and media was carefully removed. Cell pellets were snap frozen and stored at −80°C until needed. Pellets were thawed, resuspended in lysis buffer and RNA extracted using the RNeasy Mini Kit (Qiagen) according to manufacturer’s instructions. RNA concentrations were measured on a Nanodrop and 2 μg of RNA was used to generate complementary DNA (cDNA) using the Quantitech reverse transcription kit (Qiagen). Four μL of diluted cDNA was mixed with 5 μL of TaqMan master mix, 0.5 μL of nuclease free water and 0.5 μL of PXDN TaqMan probes (Mm00625468_m1). GAPDH TaqMan probes (Mm99999915_g1) were used as housekeeping controls. PCR was then performed on a QuantStudio7 real-time PCR system using standard cycling settings as below. PCR data was analyzed using the comparative CT method with the formula 2ˆ(-(ΔCT – (Average of reference ΔCT values))), as previously described.^81^ Three biological replicates were prepared for each sample, and three technical replicates were performed for each biological replicate. shScr CAF samples were used as the reference cell line in knockdown studies, while WT CAFs were used as the reference cell line in overexpression studies.

**Table:** 

Stage	Temperature (°C)	Time (mm:ss)
Hold	50	2:00
Hold	95	10:00
Cycle (40 Cycles)	95	0:15
	60	1:00
Hold	4	∞

#### Proliferation assays

For the Alamar blue assay, PXDN knockdown and overexpression CAFs and control lines were seeded at a density of 750 cells/100 μL per well in a 96-well plate. Alamar blue (1:100 in growth media) was added to cells at each time point (0, 1, 2, 3, 4, 5 and 6 days following seeding) and incubated at 37°C in a humidified incubator at 21% O_2_ supplemented with 5% CO_2_ for 2 h before being read on a FLUOstar Omega plate reader (BMG Labtech) (Ex: 544 nm, Em: 590-600 nm). For day 0 readings, Alamar blue was added to cells 1 h after initial seeding of cells into wells. Fluorescence readings from control wells without cells were subtracted from the reading of all wells with cells prior to normalisation to day 0 readings. For cell counts, PXDN knockdown and overexpression CAFs and control lines were seeded at a density of 3,000 cells/1 mL per well in a 24-well plate. At each time point, four wells were trypsinised and cells collected and counted for each cell line. Cells were pelleted at 300g for 5 min and resuspended in 200 μl before cells were counted using the Countess II cell counter.

#### Spheroid growth assay

PXDN knockdown and overexpression CAFs and controls were seeded at 20,000 CAFs per well in a 96-well low attachment round bottom tissue culture plate in 100 μL of culture media supplemented with 0.01% methylcellulose and allowed to aggregate into spheroids. Spheroids were then cultured at 37°C in a humidified incubator at 21% O_2_ supplemented with 5% CO_2_ for 16 days with imaging every 24 h using 10x magnification on the IncuCyte S3 cell imager (ESSEN BioScience). Media was changed on days 4 and 8 by careful removal of 80 μL of media per well, being careful not to disturb the spheroid, and replacing with new media. Spheroid area was calculated using the Sartorius IncuCyte Spheroid Analysis Software Module.

#### Cell morphology and spreading

PXDN knockdown, overexpression and control PyMT CAFs were seeded at a density 6 x 10^5^ cells/well in 24-well gelatin coated plates. Images of cells were taken at 10x magnification hourly for 10 h to measure cell spreading using an IncuCyte S3 cell imager (ESSEN BioScience), with a final image taken at 24 h. Wells were imaged with the IncuCyte S3 cell imager (ESSEN BioScience) every 30 min for 48 h, starting 30 min after cell seeding.

For cell morphology, images of CAFs 24 h after seeding were manually traced and ImageJ measurements of cell area, perimeter, roundness (4∗area/(π∗major axisˆ2)) and maximum caliper (the longest distance between any two points on the cell perimeter) were recorded. For cell spreading, cells were visually inspected and the number of cells with spread morphology (defined by cells with a flattened appearance and filipodia/spindle like projections from the cells body) and spherical morphology were counted within each image. This was used to calculate the percentage of total cells with spread morphology at each time point. Three images per time point, each from different wells, were analyzed for each condition in each of three biological replicates.

#### Collagen I extraction

Collagen I was prepared from rats tails as previously described.^58^ Tendons were extracted from rat tails and collagen I was solubilised in 0.5M acetic acid for 2–3 days at 4°C. Sheath and debris were removed from the collagen solution by first filtering through Wypall sheets and then centrifugation at 15,400xg at 4°C for 1 h. Supernatant was collected and collagen I was precipitated out of solution by addition of NaCl to a final concentration of 10% w/v. Precipitated collagen was collected by centrifuging solution at 15,400xg for 30 min at 4°C and discarding the supernatant. Collagen I was then re-dissolved in 0.25M acetic acid and dialyzed to 17.4 mM acetic acid with BioDesignDialysis tubing (3.5K molecular weight cut off, BioDesign Inc.). Any additional salt and debris were removed by centrifuging dialyzed collagen at 30,000xg for 1.5 h at 4°C and collecting the supernatant, before UV sterilising the collagen on ice for 20 min. Collagen concentration was measured using the Sircol assay (Biocolor life science assays) as per the manufacturer’s protocol. The collagen concentration was then adjusted to 3 mg/mL in 17.4 mM acetic acid for use in collagen contraction assays.

#### Collagen contraction assay

PXDN knockdown, overexpression and control PyMT CAFs (1.4 x 10^5^ CAFs per mL, 3.5 x 10^5^ CAFs per gel) were seeded into collagen matrices in a 6-well plate at a final concentration of 1 mg/mL collagen I, with 10% FBS and 10% collagen neutralising buffer (5% 10x MEM, 3% (w/v) NaHCO_3_, 0.1M HEPES). 2.5 mL of collagen solution containing CAFs was added per well and gels were set by incubation at 37°C for 15 min. Upon gelation, 2.5 mL complete media was added on top, and gels were detached from the edges of the wells by gently running a pipette tip between the edge of the well and the gel. Collagen gels were then cultured for 12 days at 37°C in a humidified incubator at 21% O_2_ supplemented with 5% CO_2_ with media changes every 3 days. Contraction of gels was imaged once a day using an Epson V370 photo scanner with scale. Images were imported into ImageJ (Version 1.54f) and the perimeter of the gels outlined and used to calculate the area of the gel.

#### Cell derived matrices (CDM) generation

Tissue culture plates were coated in sterile 0.2% gelatin. Gelatin coated tissue culture plates were equilibrated with media for 1 h at 37°C before cell seeding. PXDN knockdown, overexpression and control PyMT CAFs were seeded at a density of 4 x 10^4^ cells/well for 96-well plates used for proliferation studies or 6x10^5^ cells/well for 24-well plates used for cell motility studies. Starting 24 h after seeding, media was replaced every 2 days with culture media containing 50 μg/mL ascorbic acid. On day 7, plates were incubated with denuding buffer (0.5% Triton X-100, 1% Sodium Deoxycholate, 20 mM NH_4_OH in PBS) for 2 min before gently being rinsed 2x with PBS. DNAse buffer (10 μg/mL DNAse I, 5 mM MgCl_2_ in PBS) was added to wells and incubated at 37°C for 30 min before CDMs were washed 2x thoroughly with PBS and stored at 4°C in PBS +1% penicillin-Streptomycin until use.

#### Cancer cell motility on CDMs

CDMs were visually inspected with a light microscope to ensure CDMs remained present and intact prior to seeding of cancer cells. CDMs were equilibrated with PyMT 20065 medium for 1h at 37°C before cell seeding. PyMT 20065 cancer cells were seeded onto CDMs at a density of 10,000 cells/well in 24-well plates. Wells were imaged with the IncuCyte S3 cell imager (ESSEN BioScience) every 30 min for 48 h, starting 30 min after cell seeding. Cell motility was measured by tracing the movement of cells from the point of one cell division until the next cell division using the Manual Tracking with TrackMate plugin^82^ in ImageJ (Version 1.54f). To avoid differences in cell spreading at earlier time points, cells were only tracked between 24 and 48 h after seeding. Five cells per image in a total of 3 images per condition were imaged for each of 3 biological replicates, leading to a total of 45 tracked cells per condition.

#### Amplex red activity assay

A modified Amplex red assay was developed based for the detection of PXDN based on the previously designed assay described by Pape et al.^62^ WT CAFs were seeded in a 96-well plate at a density of 600 cells per well and cultured for 5 days. Before the assay, cells were washed 1x with H-medium (145 mM NaCl, 5 mM KCl, 1 mM MgCl_2_, 0.8 mM CaCl_2_, 5 mM Glucose, 10 mM HEPES) and covered with 70 μL H-medium supplemented with varying concentrations of AZD5904 per well. Cells were incubated at 37°C for 30 min before 10 μL of Amplex red was added to wells to a final concertation of 50 μM and incubated at room temperature for 5 min, followed by addition of 10 μL of NaBr to a final concentration of 25 mM and a further 5 min incubation. Finally, 10 μL of hydrogen peroxide was added to a final concentration of 1 mM per well and the assay was allowed to continue for 1 h. Amplex red fluorescence was read using a BMG Labtech ClarioStar plate reader (Ex: 577-578 nm, Em: 613-620 nm). Fluorescence readings from control conditions in which no NaBr was added used to determine PXDN specific activity readings.

#### Orthotopic cancer model with PXDN manipulated CAFs

Female FVB/JAusb mice were obtained from the Australian BioResources facility. When mice were 11 weeks of age, 1 x 10^6^ PyMT cancer cells and 3 x 10^6^ PyMT CAFs (either wild type, or containing shRNA scrambled construct, PXDN knockdown construct or overexpression of recombinant PXDN) were prepared in 50 μL of Hanks Balanced Salt Solution and injected into the right 4^th^ mammary fatpad (10 mice per group). Tumor growth was measured 3x weekly by taking length and width measurements with digital calipers and estimating tumor size with the equation: tumor volume = 0.52 x width x length^2^. Once tumors reached 520 mm^3^ in size (maximum allowed under animal ethics), mice were considered to have reached endpoint and were euthanised, and tumors were removed and weighed. Tumors were then fixed in 10% buffered formalin for 48 h, before moving to 80% ethanol for short term storage and then being passed through a graded series of increasing ethanol (70–100%) and embedded in paraffin. Lungs of mice were inflated using Fekete’s solution (58% Ethanol, 2.96% formaldehyde, 4% acetic acid in water) and then fixed in 10% buffered formalin for 48 h, before moving to 80% ethanol for short term storage and then being passed through a graded series of increasing ethanol (70–100%) and embedded in paraffin. Kaplan-Meier plots of survival and Log Rank *p*-values were generated in R (version 4.0.4, RStudio version 1.4.1103) using the Survminer package (version 0.5.0).

#### Orthotopic cancer models treated with AZD5904

AZD5904 for *in vitro* activity assays was purchased from Sapphire Bioscience (Cat # BS-82781C). AZD5904 for *in vivo* studies was provided free of charge via the AstraZeneca OpenInnovation program. Female FVB/JAusb mice were obtained from the Australian Bio Resources facility. When mice were 11 weeks of age, 1 x 10^6^ PyMT cancer cells and 3 x 10^6^ PyMT CAFs (with shRNA scrambled construct) were prepared in 50 μL of Hanks Balanced Salt Solution and injected into the right 4^th^ mammary fatpad. Tumor growth was measured as above. After tumors reached a detectable size of 50 mm^3^, mice were treated with 75 mg/kg of AZD5904 or vehicle (0.5% HPMC, 0.1% Tween80 in purified water) by oral gavage twice daily at 8a.m. and 6p.m. until tumors reached a maximum ethical size of 520 mm^3^. Upon euthanasia of mice, blood was collected by cardiac puncture into an EDTA coated collection tube and delivered to the University of Sydney Veterinary Pathology Diagnostic Services for whole blood analysis. Tumors and lungs were removed and final tumor weight recorded as above.

#### Lung metastases analysis

Sections measuring 4 μm were cut from formalin fixed, paraffin embedded lungs of mice and mounted onto superfrost plus adhesive slides. Slides were then stained using the Leica autostainer (ST5010) with Haematoxylin (Australian Biostain Haematoxylin Harris non-toxic, acidified) and Eosin Phloxine Alcoholic 1% (Australian Biostain). Once stained, sections were digitised using the Nanozoomer S210 (SDR Scientific). Metastases were identified and traced in QuPath (Version 0.3.2) to identify number of metastases per mm^2^ of lung tissue and average size of metastases. Metastatic burden was determined by calculating the percentage of lung area occupied by metastatic lesions.

#### Alpha-smooth muscle actin IHC analysis

IHC staining was performed on formalin fixed, paraffin embedded tumor tissues from mouse studies. Tumor sections measuring 4 μm were baked onto Superfrost plus slides (ThermoFisher Scientific, 491PLUS4). Sections were then deparaffinised in Bond Dewax (AR9222) + 100% ethanol. Heat induced epitope retrieval was performed at pH 9 (Leica, AR9640) for 30 min at 100°C. α-SMA (1:100, Abcam, ab5694) staining was performed using the Bond RX Austostainer (Leica) using the Bond Polymer Refine Detection kit (Leica, DS9800) with an antibody incubation of 60 min. Slides were counterstained with Haematoxylin (Australian Biostain Haematoxylin Harris non-toxic, acidified) and cover slipped.

Slides were digitised using the using the Nanozoomer S210 (SDR Scientific). IHC analysis was performed in QuPath (Version 0.3.2). For α-SMA analysis in the PXDN knockdown study, DAB vector values were set to {0.269 0.564 0.778} and Hematoxylin vector values were set to {0.269 0.586 0.778}. A thresholder was created to detect α-SMA positive staining using a Gaussian pre-filter and a threshold of 0.6 with a smoothing sigma of 0.5. For a-SMA analysis in the PXDN overexpression study, DAB vector values were set to {0.269 0.568 0.778} and Hematoxylin vector values were set to {0.593 0.695 0.406}. A thresholder was created to detect α-SMA positive staining using a Gaussian pre-filter and a threshold of 0.5 with a smoothing sigma of 0.25. Thresholders were used to calculate the percentage of tissue with positive staining.

### Quantification and statistical analysis

Data imputation was performed in RStudio (Version 1.4.1103) using R version 4.0.4. For survival analysis, log Rank *p*-values were calculated using the Survminer package (version 0.5.0) in R-Studio (Version 1.4.1103, R version 4.0.4) and were considered significant if *p* < 0.05. For comparisons, unpaired Student’s t-tests were used with GraphPad Prism 10. *p*-values were considered significant if *p* < 0.05. As noted in the figure legends, error bars represent the standard error of the mean when six or more technical replicates were averaged per biological replicate, and the mean of these technical replicates is shown in the figures. In cases where all technical replicates across all biological replicates are displayed, error bars represent the standard deviation.

## Supplemental information

Document S1. Figures S1–S10, and Tables S1–S4, and Data S1/Methods S1

## References

[1] Siegel R.L., Miller K.D., Jemal A. (2020). Cancer statistics, 2020. CA Cancer J. Clin..

[2] Chikara B.S., Parang K. (2023). Global Cancer Statistics 2022: the trends projection analysis. Chem. Biol. Lett..

[3] Giaquinto A.N., Sung H., Miller K.D., Kramer J.L., Newman L.A., Minihan A., Jemal A., Siegel R.L. (2022). Breast cancer statistics, 2022. CA Cancer J. Clin..

[4] Pan H., Gray R., Braybrooke J., Davies C., Taylor C., McGale P., Peto R., Pritchard K.I., Bergh J., Dowsett M. (2017). 20-Year Risks of Breast-Cancer Recurrence after Stopping Endocrine Therapy at 5 Years. N. Engl. J. Med..

[5] Dowsett M., Cuzick J., Ingle J., Coates A., Forbes J., Bliss J., Buyse M., Baum M., Buzdar A., Colleoni M. (2010). Meta-analysis of breast cancer outcomes in adjuvant trials of aromatase inhibitors versus tamoxifen. J. Clin. Oncol..

[6] Pedersen R.N., Esen B.Ö., Mellemkjær L., Christiansen P., Ejlertsen B., Lash T.L., Nørgaard M., Cronin-Fenton D. (2022). The Incidence of Breast Cancer Recurrence 10-32 Years After Primary Diagnosis. J. Natl. Cancer Inst..

[7] Xi G., Qiu L., Xu S., Guo W., Fu F., Kang D., Zheng L., He J., Zhang Q., Li L. (2021). Computer-assisted quantification of tumor-associated collagen signatures to improve the prognosis prediction of breast cancer. BMC Med..

[8] Xi G., Guo W., Kang D., Ma J., Fu F., Qiu L., Zheng L., He J., Fang N., Chen J. (2021). Large-scale tumor-associated collagen signatures identify high-risk breast cancer patients. Theranostics.

[9] Esbona K., Yi Y., Saha S., Yu M., Van Doorn R.R., Conklin M.W., Graham D.S., Wisinski K.B., Ponik S.M., Eliceiri K.W. (2018). The Presence of Cyclooxygenase 2, Tumor-Associated Macrophages, and Collagen Alignment as Prognostic Markers for Invasive Breast Carcinoma Patients. Am. J. Pathol..

[10] Conklin M.W., Eickhoff J.C., Riching K.M., Pehlke C.A., Eliceiri K.W., Provenzano P.P., Friedl A., Keely P.J. (2011). Aligned collagen is a prognostic signature for survival in human breast carcinoma. Am. J. Pathol..

[11] Provenzano P.P., Eliceiri K.W., Campbell J.M., Inman D.R., White J.G., Keely P.J. (2006). Collagen reorganization at the tumor-stromal interface facilitates local invasion. BMC Med..

[12] Micke P., Strell C., Mattsson J., Martín-Bernabé A., Brunnström H., Huvila J., Sund M., Wärnberg F., Ponten F., Glimelius B. (2021). The prognostic impact of the tumour stroma fraction: A machine learning-based analysis in 16 human solid tumour types. EBioMedicine.

[13] Chitty J.L., Cox T.R. (2025). The extracellular matrix in cancer: from understanding to targeting. Trends in Cancer.

[14] Lepucki A., Orlińska K., Mielczarek-Palacz A., Kabut J., Olczyk P., Komosińska-Vassev K. (2022). The role of extracellular matrix proteins in breast cancer. J. Clin. Med..

[15] Mehraj U., Dar A.H., Wani N.A., Mir M.A. (2021). Tumor microenvironment promotes breast cancer chemoresistance. Cancer Chemother. Pharmacol..

[16] Miroshnychenko D., Miti T., Kumar P., Miller A., Laurie M., Giraldo N., Bui M.M., Altrock P.M., Basanta D., Marusyk A. (2023). Stroma-Mediated Breast Cancer Cell Proliferation Indirectly Drives Chemoresistance by Accelerating Tumor Recovery between Chemotherapy Cycles. Cancer Res..

[17] Di Martino J.S., Akhter T., Bravo-Cordero J.J. (2021). Remodeling the ECM: implications for metastasis and tumor dormancy. Cancers (Basel).

[18] Bhattacharjee S., Hamberger F., Ravichandra A., Miller M., Nair A., Affo S., Filliol A., Chin L., Savage T.M., Yin D. (2024). Tumor restriction by type I collagen opposes tumor-promoting effects of cancer-associated fibroblasts. J. Clin. Investig..

[19] Kim S.H., Lee H.Y., Jung S.P., Kim S., Lee J.E., Nam S.J., Bae J.W. (2014). Role of secreted type I collagen derived from stromal cells in two breast cancer cell lines. Oncol. Lett..

[20] Nolan J., Mahdi A.F., Dunne C.P., Kiely P.A. (2020). Collagen and fibronectin promote an aggressive cancer phenotype in breast cancer cells but drive autonomous gene expression patterns. Gene.

[21] Whatcott C.J., Han H., Von Hoff D.D. (2015). Orchestrating the tumor microenvironment to improve survival for patients with pancreatic cancer: normalization, not destruction. Cancer J..

[22] Lázár E., Péterfi Z., Sirokmány G., Kovács H.A., Klement E., Medzihradszky K.F., Geiszt M. (2015). Structure-function analysis of peroxidasin provides insight into the mechanism of collagen IV crosslinking. Free Radic. Biol. Med..

[23] Ero-Tolliver I.A., Hudson B.G., Bhave G. (2015). The Ancient Immunoglobulin Domains of Peroxidasin Are Required to Form Sulfilimine Cross-links in Collagen IV. J. Biol. Chem..

[24] Bhave G., Cummings C.F., Vanacore R.M., Kumagai-Cresse C., Ero-Tolliver I.A., Rafi M., Kang J.S., Pedchenko V., Fessler L.I., Fessler J.H., Hudson B.G. (2012). Peroxidasin forms sulfilimine chemical bonds using hypohalous acids in tissue genesis. Nat. Chem. Biol..

[25] Wyllie K., Panagopoulos V., Cox T.R. (2023). The role of peroxidasin in solid cancer progression. Biochem. Soc. Trans..

[26] Zhou X., Sun Q., Xu C., Zhou Z., Chen X., Zhu X., Huang Z., Wang W., Shi Y. (2022). A systematic pan-cancer analysis of PXDN as a potential target for clinical diagnosis and treatment. Front. Oncol..

[27] Young C.D., Zimmerman L.J., Hoshino D., Formisano L., Hanker A.B., Gatza M.L., Morrison M.M., Moore P.D., Whitwell C.A., Dave B. (2015). Activating PIK3CA Mutations Induce an Epidermal Growth Factor Receptor (EGFR)/Extracellular Signal-regulated Kinase (ERK) Paracrine Signaling Axis in Basal-like Breast Cancer. Mol. Cell. Proteomics.

[28] Wang Q., Karvelsson S.T., Kotronoulas A., Gudjonsson T., Halldorsson S., Rolfsson O. (2022). Glutamine-Fructose-6-Phosphate Transaminase 2 (GFPT2) Is Upregulated in Breast Epithelial-Mesenchymal Transition and Responds to Oxidative Stress. Mol. Cell. Proteomics.

[29] Graf F., Horn P., Ho A.D., Boutros M., Maercker C. (2021). The extracellular matrix proteins type I collagen, type III collagen, fibronectin, and laminin 421 stimulate migration of cancer cells. FASEB J..

[30] Sigurdardottir A.K., Jonasdottir A.S., Asbjarnarson A., Helgudottir H.R., Gudjonsson T., Traustadottir G.A. (2021). Peroxidasin enhances basal phenotype and inhibits branching morphogenesis in breast epithelial progenitor cell line D492. J. Mammary Gland Biol. Neoplasia.

[31] Papanicolaou M., Parker A.L., Yam M., Filipe E.C., Wu S.Z., Chitty J.L., Wyllie K., Tran E., Mok E., Nadalini A. (2022). Temporal profiling of the breast tumour microenvironment reveals collagen XII as a driver of metastasis. Nat. Commun..

[32] Fluck M.M., Schaffhausen B.S. (2009). Lessons in signaling and tumorigenesis from polyomavirus middle T antigen. Microbiol. Mol. Biol. Rev..

[33] Attalla S., Taifour T., Bui T., Muller W. (2021). Insights from transgenic mouse models of PyMT-induced breast cancer: recapitulating human breast cancer progression in vivo. Oncogene.

[34] Lin E.Y., Jones J.G., Li P., Zhu L., Whitney K.D., Muller W.J., Pollard J.W. (2003). Progression to malignancy in the polyoma middle T oncoprotein mouse breast cancer model provides a reliable model for human diseases. Am. J. Pathol..

[35] Naba A., Clauser K.R., Hoersch S., Liu H., Carr S.A., Hynes R.O. (2012). The matrisome: in silico definition and in vivo characterization by proteomics of normal and tumor extracellular matrices. Mol. Cell. Proteomics.

[36] Cox T.R. (2021). The matrix in cancer. Nat. Rev. Cancer.

[37] Kaushik S., Pickup M.W., Weaver V.M. (2016). From transformation to metastasis: deconstructing the extracellular matrix in breast cancer. Cancer Metastasis Rev..

[38] Najafi M., Farhood B., Mortezaee K. (2019). Extracellular matrix (ECM) stiffness and degradation as cancer drivers. J. Cell. Biochem..

[39] Oskarsson T. (2013). Extracellular matrix components in breast cancer progression and metastasis. Breast.

[40] Cai Y., Nogales-Cadenas R., Zhang Q., Lin J.R., Zhang W., O'Brien K., Montagna C., Zhang Z.D. (2017). Transcriptomic dynamics of breast cancer progression in the MMTV-PyMT mouse model. BMC Genom..

[41] Engstrøm M.J., Opdahl S., Hagen A.I., Romundstad P.R., Akslen L.A., Haugen O.A., Vatten L.J., Bofin A.M. (2013). Molecular subtypes, histopathological grade and survival in a historic cohort of breast cancer patients. Breast Cancer Res. Treat..

[42] Cid S., Eiro N., Fernández B., Sánchez R., Andicoechea A., Fernández-Muñiz P.I., González L.O., Vizoso F.J. (2018). Prognostic influence of tumor stroma on breast cancer subtypes. Clin. Breast Cancer.

[43] Valdés-Mora F., Salomon R., Gloss B.S., Law A.M.K., Venhuizen J., Castillo L., Murphy K.J., Magenau A., Papanicolaou M., Rodriguez de la Fuente L. (2021). Single-cell transcriptomics reveals involution mimicry during the specification of the basal breast cancer subtype. Cell Rep..

[44] Tarhan L., Bistline J., Chang J., Galloway B., Hanna E., Weitz E. (2023). Single Cell Portal: an interactive home for single-cell genomics data. bioRxiv.

[45] Wu S.Z., Al-Eryani G., Roden D.L., Junankar S., Harvey K., Andersson A., Thennavan A., Wang C., Torpy J.R., Bartonicek N. (2021). A single-cell and spatially resolved atlas of human breast cancers. Nat. Genet..

[46] Dougan J., Hawsawi O., Burton L.J., Edwards G., Jones K., Zou J., Nagappan P., Wang G., Zhang Q., Danaher A. (2019). Proteomics-Metabolomics Combined Approach Identifies Peroxidasin as a Protector against Metabolic and Oxidative Stress in Prostate Cancer. Int. J. Mol. Sci..

[47] Zheng Y.-Z., Liang L. (2018). High expression of PXDN is associated with poor prognosis and promotes proliferation, invasion as well as migration in ovarian cancer. Ann. Diagn. Pathol..

[48] Tian Y., Qiu S., Yang S., Jiang Y., Hu H., Yang C., Cao J., Chen S., Hao M., Li H., Zhu J. (2024). The oncogenic role and prognostic value of PXDN in human stomach adenocarcinoma. BMC Cancer.

[49] Hanmer K.L., Mavri-Damelin D. (2018). Peroxidasin is a novel target of the redox-sensitive transcription factor Nrf2. Gene.

[50] Sitole B.N., Mavri-Damelin D. (2018). Peroxidasin is regulated by the epithelial-mesenchymal transition master transcription factor Snai1. Gene.

[51] Cao J., Zhang G., Liu Z., Xu Q., Li C., Cheng G., Shi R. (2021). Peroxidasin promotes diabetic vascular endothelial dysfunction induced by advanced glycation end products via NOX2/HOCl/Akt/eNOS pathway. Redox Biol..

[52] Lee S.-W., Kim H.K., Naidansuren P., Ham K.A., Choi H.S., Ahn H.Y., Kim M., Kang D.H., Kang S.W., Joe Y.A. (2020). Peroxidasin is essential for endothelial cell survival and growth signaling by sulfilimine crosslink-dependent matrix assembly. FASEB J..

[53] Dempsey B., Cruz L.C., Mineiro M.F., da Silva R.P., Meotti F.C. (2022). Uric Acid Reacts with Peroxidasin, Decreases Collagen IV Crosslink, and Impairs Human Endothelial Cell Migration and Adhesion. Antioxidants.

[54] Bathish B., Turner R., Paumann-Page M., Kettle A.J., Winterbourn C.C. (2018). Characterisation of peroxidasin activity in isolated extracellular matrix and direct detection of hypobromous acid formation. Arch. Biochem. Biophys..

[55] Yao L., Zhou Y., Li J., Wickens L., Conforti F., Rattu A., Ibrahim F.M., Alzetani A., Marshall B.G., Fletcher S.V. (2021). Bidirectional epithelial-mesenchymal crosstalk provides self-sustaining profibrotic signals in pulmonary fibrosis. J. Biol. Chem..

[56] Péterfi Z., Donkó A., Orient A., Sum A., Prókai A., Molnár B., Veréb Z., Rajnavölgyi E., Kovács K.J., Müller V. (2009). Peroxidasin is secreted and incorporated into the extracellular matrix of myofibroblasts and fibrotic kidney. Am. J. Pathol..

[57] Calvo F., Ege N., Grande-Garcia A., Hooper S., Jenkins R.P., Chaudhry S.I., Harrington K., Williamson P., Moeendarbary E., Charras G., Sahai E. (2013). Mechanotransduction and YAP-dependent matrix remodelling is required for the generation and maintenance of cancer-associated fibroblasts. Nat. Cell Biol..

[58] Chitty J.L., Skhinas J.N., Filipe E.C., Wang S., Cupello C.R., Grant R.D., Yam M., Papanicolaou M., Major G., Zaratzian A. (2020). The Mini-Organo: A rapid high-throughput 3D coculture organotypic assay for oncology screening and drug development. Cancer Rep..

[59] Franco-Barraza J., Beacham D.A., Amatangelo M.D., Cukierman E. (2016). Preparation of extracellular matrices produced by cultured and primary fibroblasts. Curr. Protoc. Cell Biol..

[60] Luong T., Cukierman E. (2022). Eribulin normalizes pancreatic cancer-associated fibroblasts by simulating selected features of TGFβ inhibition. BMC Cancer.

[61] Zeneca A. (2024). Preclinical Molecules - AZD5904. https://openinnovation.astrazeneca.com/preclinical-research/preclinical-molecules/azd59041.html.

[62] Pape V.F.S., Kovács H.A., Szatmári I., Ugrai I., Szikora B., Kacskovics I., May Z., Szoboszlai N., Sirokmány G., Geiszt M. (2022). Measuring peroxidasin activity in live cells using bromide addition for signal amplification. Redox Biol..

[63] Sevcnikar B., Schaffner I., Chuang C.Y., Gamon L., Paumann-Page M., Hofbauer S., Davies M.J., Furtmüller P.G., Obinger C. (2020). The leucine-rich repeat domain of human peroxidasin 1 promotes binding to laminin in basement membranes. Arch. Biochem. Biophys..

[64] Johnston K.A., Lopez K.M. (2018). Lysyl oxidase in cancer inhibition and metastasis. Cancer Lett..

[65] Setargew Y.F.I., Wyllie K., Grant R.D., Chitty J.L., Cox T.R. (2021). Targeting Lysyl Oxidase Family Meditated Matrix Cross-Linking as an Anti-Stromal Therapy in Solid Tumours. Cancers (Basel).

[66] Jabłońska-Trypuć A., Matejczyk M., Rosochacki S. (2016). Matrix metalloproteinases (MMPs), the main extracellular matrix (ECM) enzymes in collagen degradation, as a target for anticancer drugs. J. Enzyme Inhib. Med. Chem..

[67] Popova N.V., Jücker M. (2022). The functional role of extracellular matrix proteins in cancer. Cancers (Basel).

[68] Smith-Díaz C.C., Kumar A., Das A., Pace P., Chitcholtan K., Magon N.J., Hossain S.M., Eccles M.R., Winterbourn C.C., Paumann-Page M. (2025). Peroxidasin is associated with a mesenchymal-like transcriptional phenotype and promotes invasion in metastatic melanoma. Free Radic. Biol. Med..

[69] Jayachandran A., Prithviraj P., Lo P.H., Walkiewicz M., Anaka M., Woods B.L., Tan B., Behren A., Cebon J., McKeown S.J. (2016). Identifying and targeting determinants of melanoma cellular invasion. Oncotarget.

[70] Panagopoulos V., Leach D.A., Zinonos I., Ponomarev V., Licari G., Liapis V., Ingman W.V., Anderson P., DeNichilo M.O., Evdokiou A. (2017). Inflammatory peroxidases promote breast cancer progression in mice via regulation of the tumour microenvironment. Int. J. Oncol..

[71] Rymaszewski A.L., Tate E., Yimbesalu J.P., Gelman A.E., Jarzembowski J.A., Zhang H., Pritchard K.A., Vikis H.G. (2014). The role of neutrophil myeloperoxidase in models of lung tumor development. Cancers (Basel).

[72] Williams C., Noll J.E., Harnas D., Parkinson H.B., Hewett D.R., Zannettino A.C.W., Vandyke K., Cox T.R. (2026). Therapeutic inhibition of myeloperoxidase with AZD5904 attenuates disease progression in mouse models of early-stage and relapsed multiple myeloma. Vasilios Panagopoulos. Haematologica.

[73] Williams C.M.D., Noll J.E., Bradey A.L., Duggan J., Wilczek V.J., Masavuli M.G., Grubor-Bauk B., Panagopoulos R.A., Hewett D.R., Mrozik K.M. (2023). Myeloperoxidase creates a permissive microenvironmental niche for the progression of multiple myeloma. Br. J. Haematol..

[74] Cancer Genome Atlas Network (2012). Comprehensive molecular portraits of human breast tumours. Nature.

[75] Millar E.K.A., Anderson L.R., McNeil C.M., O'Toole S.A., Pinese M., Crea P., Morey A.L., Biankin A.V., Henshall S.M., Musgrove E.A. (2009). BAG-1 predicts patient outcome and tamoxifen responsiveness in ER-positive invasive ductal carcinoma of the breast. Br. J. Cancer.

[76] Floerchinger A., Murphy K.J., Latham S.L., Warren S.C., McCulloch A.T., Lee Y.K., Stoehr J., Mélénec P., Guaman C.S., Metcalf X.L. (2021). Optimizing metastatic-cascade-dependent Rac1 targeting in breast cancer: Guidance using optical window intravital FRET imaging. Cell Rep..

[77] Shao X., Taha I.N., Clauser K.R., Gao Y.T., Naba A. (2020). MatrisomeDB: the ECM-protein knowledge database. Nucleic Acids Res..

[78] Lazar C., Gatto L., Ferro M., Bruley C., Burger T. (2016). Accounting for the Multiple Natures of Missing Values in Label-Free Quantitative Proteomics Data Sets to Compare Imputation Strategies. J. Proteome Res..

[79] Butler A., Hoffman P., Smibert P., Papalexi E., Satija R. (2018). Integrating single-cell transcriptomic data across different conditions, technologies, and species. Nat. Biotechnol..

[80] Dull T., Zufferey R., Kelly M., Mandel R.J., Nguyen M., Trono D., Naldini L. (1998). A third-generation lentivirus vector with a conditional packaging system. J. Virol..

[81] Schmittgen T.D., Livak K.J. (2008). Analyzing real-time PCR data by the comparative C(T) method. Nat. Protoc..

[82] Tinevez J.-Y., Perry N., Schindelin J., Hoopes G.M., Reynolds G.D., Laplantine E., Bednarek S.Y., Shorte S.L., Eliceiri K.W. (2017). TrackMate: An open and extensible platform for single-particle tracking. Methods.

