# Supplementary material for: Stromal peroxidasin drives early tumor growth in breast cancer
Source: iScience. 2026 Jun 1;29(6):116078. doi: 10.1016/j.isci.2026.116078 (PMC13253085; doi:10.1016/j.isci.2026.116078)
Supplement: Document S1. Figures S1–S10, and Tables S1–S4, and Data S1/Methods S1 [file mmc1.pdf]

iScience, Volume *29*

Supplemental information

Stromal peroxidasin drives early tumor growth in breast cancer

Kaitlin Wyllie, Ellie T.Y. Mok, Que Emmi Tran, Elysse C. Filipe, Jessica L. Chitty, Ron Enriquez, Anaiis Zaratzian, Andrew M. Da Silva, Michael Tayao, David Gallego-Ortega, Sandra O'Toole, Amelia L. Parker, Vasilios Panagopoulos, and Thomas R. Cox

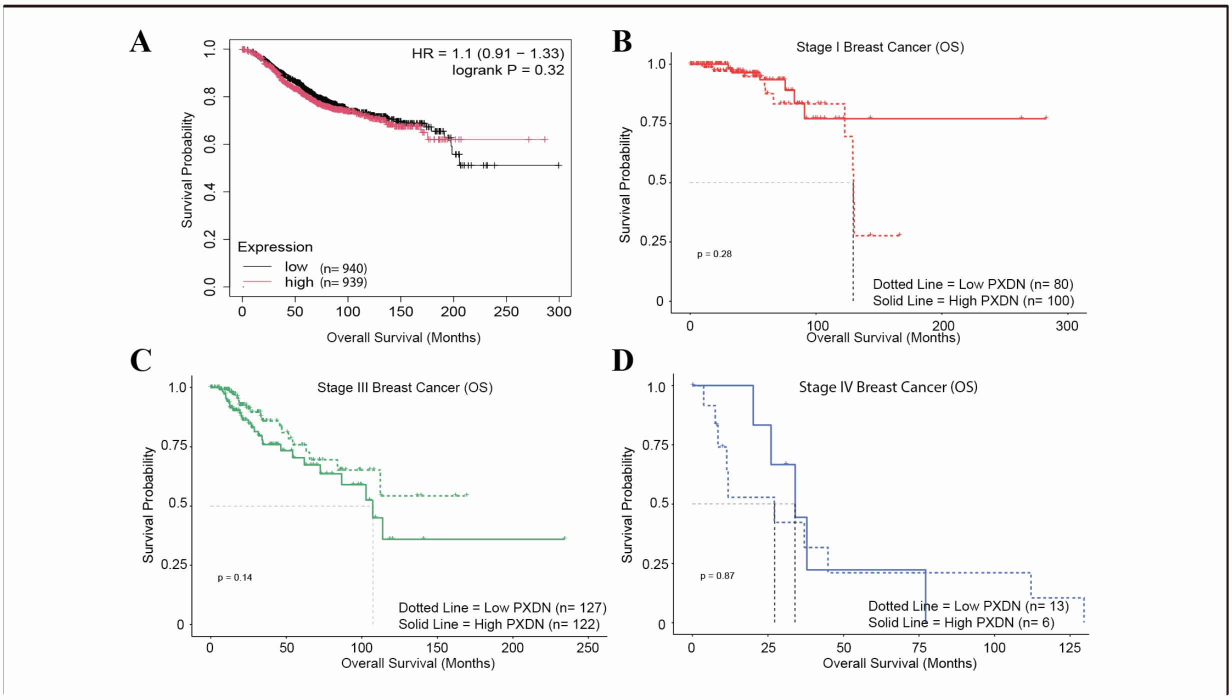

Supplementary Figure 1: Association between PXDN expression and overall survival in breast cancer. (**A**) Overall survival of 1,879 breast cancer patients stratified by median gene chip expression of PXDN expression (Affy ID 212012_at) determined by the Kaplan-Meier plotter online tool (https://kmplot.com/analysis/). The hazard ratio and confidence intervals were calculated using the Cox proportional hazard model. (**B**) Overall survival of stage I breast cancer patients (n=180) from the TCGA breast cancer cohort stratified by median mRNA expression of PXDN (dotted line = low PXDN expression, solid line = high PXDN expression). (**C**) Overall survival of stage III breast cancer patients (n=249) from the TCGA breast cancer cohort stratified by median mRNA expression of PXDN (dotted line = low PXDN expression, solid line = high PXDN expression). (**D**) Overall survival of stage IV breast cancer patients (n=19) from the TCGA breast cancer cohort stratified by median mRNA expression of PXDN (dotted line = low PXDN expression, solid line = high PXDN expression). (B-D) p-values were calculated using the Log Rank test.

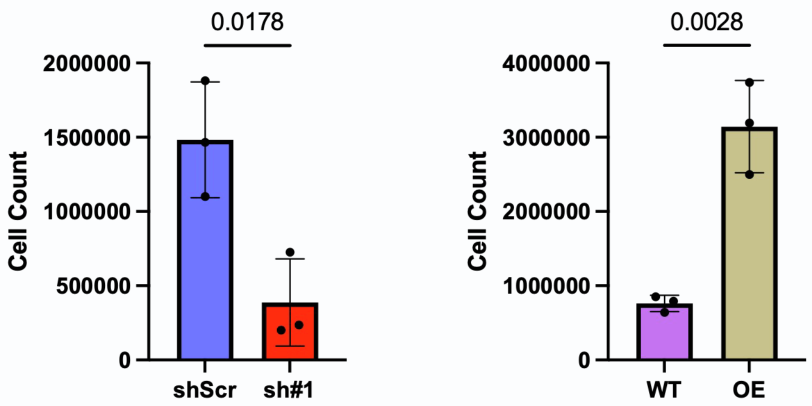

Supplementary Figure 2: Proliferation of CAFs in 2D as measured by cell count. Differences between groups were tested with a student's t-test. Data show average technical replicates for each of 3 biological replicates. (**A**) shScr and sh#1 CAF cell counts after 5 days of growth in 2D. (**B**) WT and OE CAF cell counts after 5 days of growth in 2D.

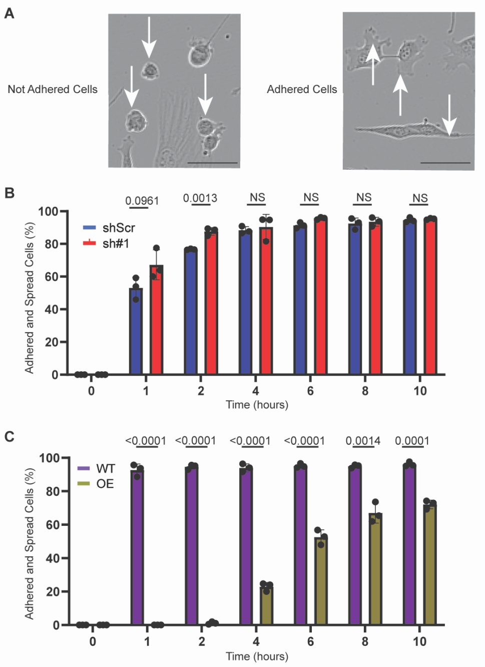

Supplementary Figure 3: CAF spreading and morphology over a 10-hour time-course following seeding onto gelatin coated 24-well plates. (**A**) Representative images of CAF spreading and morphology. CAFs were considered to have not adhered if they were spherical (arrows on the left) or spread if they had flattened, non-spherical cell bodies with spindle like filopodia structures (arrows on the right). Scale bar = 50µm. (**B**) Percentage of shScr (blue) and sh#1 (red) CAFs with spread morphology at different time points. (**C**) Percentage of WT (purple) and OE (gold) CAFs with spread morphology at different time points. All cells in a 2.25mm^2 image (minimum 250 cells per image) were counted for cell spreading analysis. Three images were analysed per time point for each CAF line. Differences between CAF lines at each time point were assessed using student's T-tests and were considered significant if $p < 0.05$.

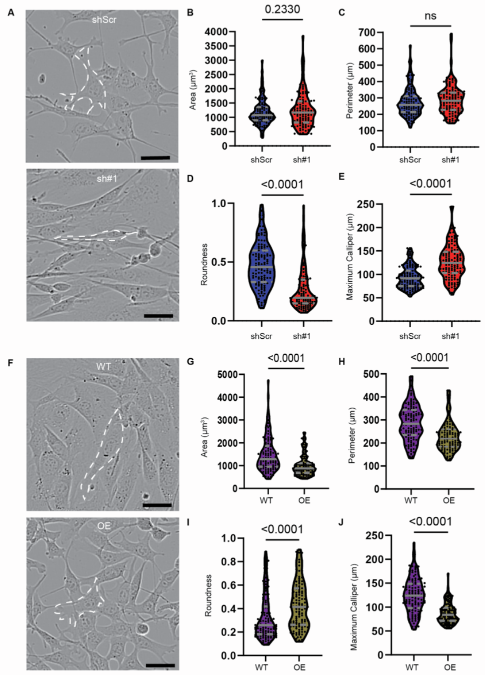

Supplementary Figure 4: Morphology of CAFs at 24 hours after seeding. (**A,F**) Representative images of shScr and sh#1 (**A**) or WT and OE (**F**) CAF morphology. An example cell is outlined in white in each image. Scale bar = 40µm. Cells were traced and ImageJ was used to measure (**B,G**) CAF surface area, (**C,H**) CAF perimeter, (**D,I**) CAF roundness (calculated with the equation 4*area/(π*major_axis^2)), and **(E,J)** maximum caliper (longest distance between any two points of the CAF perimeter) in shScr and sh#1 (**B-E**) or WT and OE (**G-J**) CAFs. Cells were measured in three biological replicates, with two images per replicate. All individual cells in each image were plotted and the median (grey line) and quartile ranges (dotted lines) then calculated. Differences were calculated with a student's t-test and considered significant if $p < 0.05$.

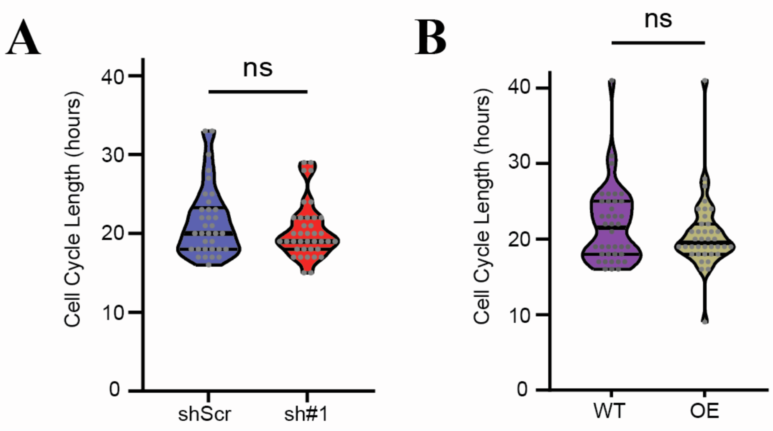

Supplementary Figure 5: Time taken for cancer cells to complete a full cell cycle (from one cell division until the next) when seeded upon matrices produced by CAFs with PXDN knockdown (sh#1) (**A**) or overexpression (OE) (**B**) and their respective controls (shScr, WT). Differences were calculated with a student's t-test and considered significant if $p < 0.05$. Median and quartile ranges (solid lines) are indicated in black.

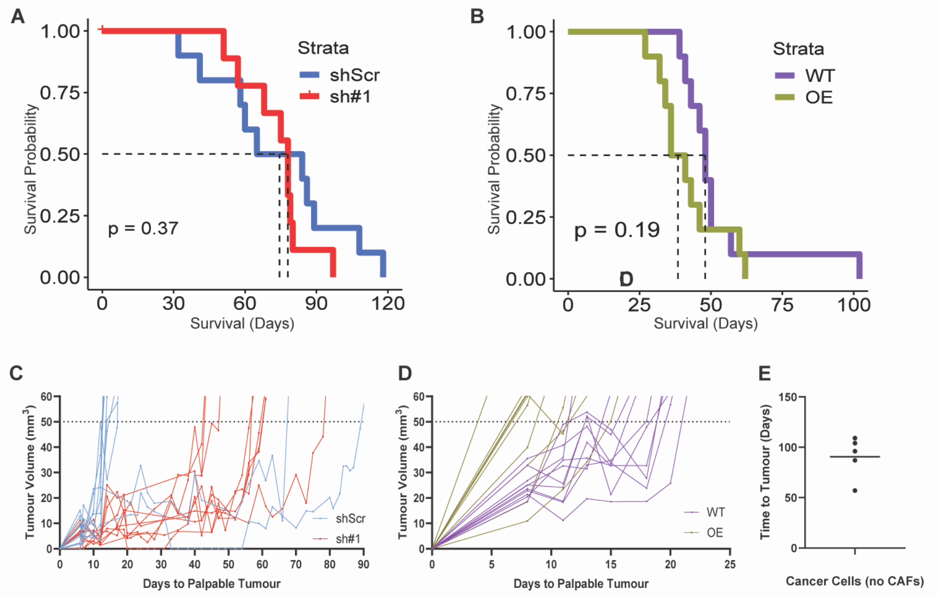

Supplementary Figure 6: Growth of tumours containing cancer cells and CAFs with PXDN manipulation. (**A, B**) Kaplan-Meier curves of survival as measured from the implantation of cancer cells with shScr or sh#1 CAFs (**A**) or with WT or OE CAFs (**B**) until tumours reached a maximum ethical size equivalent to 1cm x 1cm. P-values were calculated using the Log Rank test. (**C, D**) Tumour formation and early growth dynamics of tumours containing cancer cells with shScr or sh#1 CAFs (**C**) or with WT or OE CAFs (**D**). (**E**) Time until tumours reached a detectable size of $50mm^3$ in tumours containing $1x10^6$ cancer cells alone (without CAFs).

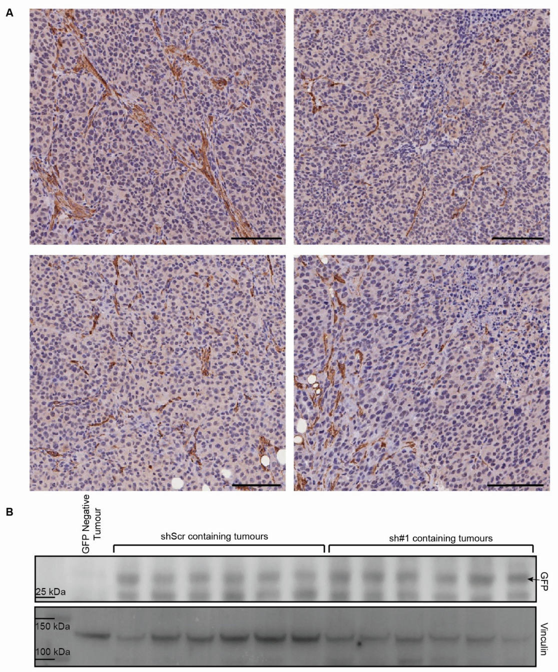

Supplementary Figure 7: CAF presence in the PyMT orthotopic model of breast cancer. (**A**) Examples of a-SMA positive IHC staining in tumours from four mice implanted with PyMT cancer cells only (no co-implanted CAFs) indicative of recruitment of a-SMA positive host fibroblasts into the developing tumours. Scale bar = 100µm. (**B**) Western blot showing presence of GFP in individual tumours from the PXDN knockdown study. Only CAFs expressing the shScr or shPXDN constructs will be positive for GFP. Vinculin was used as a housekeeping control. Tumour lysate from a tumour containing cancer cells and unmodified parental CAFs was used as a GFP negative control.

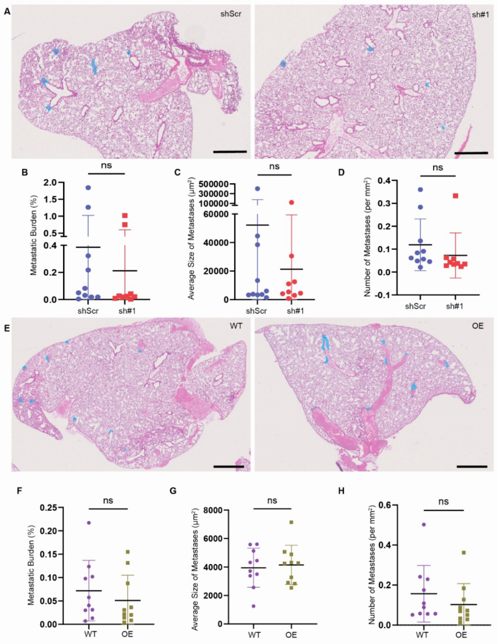

Supplementary Figure 8: Metastatic burden in mouse lungs from the orthotopic mouse models generated by co-implantation of cancer cells with shScr or sh#1 (**A-D**) or WT or OE (**E-H**) CAFs into the mammary fat pads of mice. (**A,E**) Representative images of metastases. Scale bar = 100µm. Metastases outlined in blue. (**B,F**) Metastatic burden (percentage of the lung area occupied by metastases). (**C,G**) Average size of metastases. (**D,H**) Number of metastases per mm^2 of lung tissue. Differences between groups were tested using a student's t-test and considered significant if $p < 0.05$.

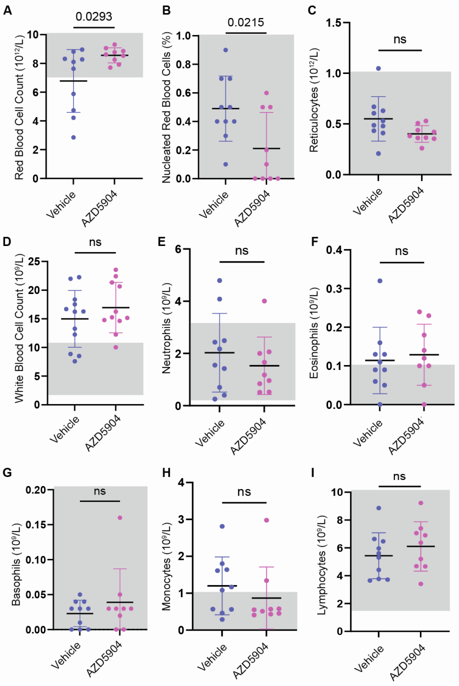

Supplementary Figure 9: Blood cell counts in whole blood analysis of the orthotopic cohort of mice treated with AZD5904 (n=9) or vehicle (n=10). The normal range for female mice are highlighted in grey. (**A**) red blood cell count. (**B**) Percentage of nucleated red blood cells. (**C**) Reticulocyte count. (**D**) Total white blood cell count. (**E**) Neutrophil count. (**F**) Eosinophil count. (**G**) Basophil count. (**H)** Monocyte count. (**I**) Lymphocyte count. Differences between groups were tested using a student's- T-test and were considered significant if $p < 0.05$.

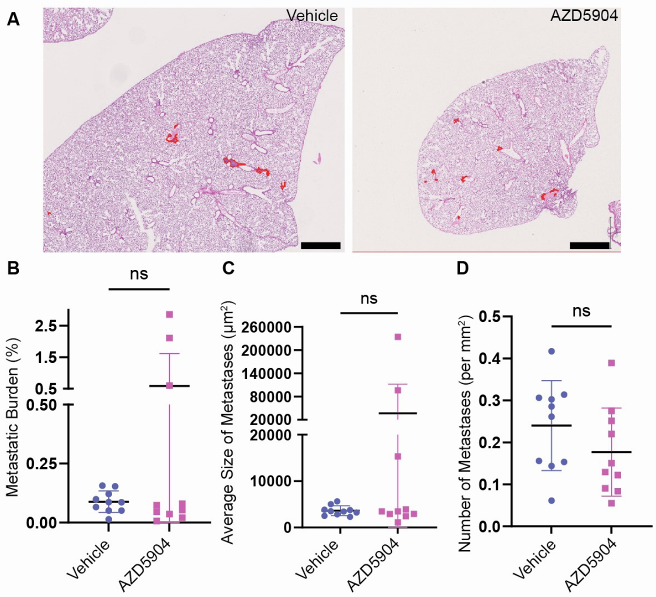

Supplementary Figure 10: Metastasis to the lungs of mice in an orthotopic model of breast cancer treated with AZD5904 (n=10) or vehicle (n=10). (**A**) Representative images of mouse lungs. Metastases outlined in red. Scale bar = 1mm. (**B**) Total Metastatic burden in lungs. (**C**) Average metastases size in μm^2. (**D**) Number of metastases per mm^2 of lung tissue. Differences between groups were tested using student's t-tests and were considered significant if $p < 0.05$.

Supplementary Table 1: Significantly differentially expressed ECM molecules in early, mid and late-stage mammary tumour samples from the PyMT mouse model of breast cancer in comparison to healthy fat pad tissue as determined by an ANOVA p-value of <0.05.

A2m	Col8a2	Lama3	S100a1
Abi3bp	Col9a1	Lama4	S100a4
Adam10	Creld2	Lama5	S100a8
Adam22	Crispld2	Lamb1	S100a9
Adam9	Cstb	Lamb2	S100b
Adamtsl4	Ctsb	Lamc1	Sdc4
Adipoq	Ctsc	Lamc2	Serpina1a;Serpina1c;Serpina1a
Agt	Ctsd	Lgals1	Serpina1b
Anxa1	Ctsh	Lgals12	Serpina1e
Anxa2	Ctss	Lgals7	Serpinb1a
Anxa6	Ctsz	Lgals8	Serpinb5
Anxa7	Dcn	Lgalsl	Serpine2
Anxa8	Dpt	Lman1	Serpinf2
Aspn	Ecm1	Loxl1	Sfrp2
Bmp1	Efemp1	Lrg1	Sparc
C1qa	Eln	Ltbp1	Sparcl1
C1qtnf3	Emid1	Ltbp2	Spp1
Cd209b	Emilin1	Ltbp3	Srpx
Chad	Emilin2	Lum	Srpx2
Cilp	F10	Matn1	St14
Clec10a	F13b	Mfap1a;Mfap1b	Sulf2
Col11a1	Fam20a	Mfap4	Svep1
Col12a1	Fam20c	Mfge8	Tgfb1
Col14a1	Fbln2	Mmp12	Tgfb2
Col15a1	Fbn1	Mmp2	Tgfb3
Col16a1	Fbn2	Muc1	Tgfbi
Col18a1	Fga	Muc15	Tgm1
Col1a1	Fgb	Nid1	Tgm2
Col1a2	Fgg	Nid2	Thbs1
Col22a1	Fn1	Npnt	Thbs2
Col3a1	Gas6	Ntn1	Thbs4
Col4a2	Gpc4;Gpc6;Gpc6	Ogn	Timp2
Col4a6	Hcfc1	P4ha2	Timp3
Col5a1	Hspg2	Papln	Tinagl1
Col5a2	Igfals	Plau	Tnc
Col5a3	Igfbp5	Plod1	Tnxb
Col6a1	Igfbp6	Plod2	Try10
Col6a2	Igfbp7	Plod3	Vcan
Col6a3	Il1rn	Plxdc2	Vtn
Col6a5	Itih2	Plxnb2	Vwa1
Col6a6	Itih3	Postn	Vwa5a
Col7a1	Itih5	Prelp	Vwf
Col8a1	Lama2	Pxdn	

Supplementary Table 2: *Demographics of patients with high and low PXDN (based on median PXDN gene expression) in the TCGA invasive breast cancer cohort. Percentages were calculated using a sum of all patients within a high or low PXDN grouping for each clinical factor.*
NOS = Not otherwise specified
Stage X = staging cannot be determined

	**PXDN High**	**PXDN Low**	**Total**
**Total Patients**	541	541	1082
**Average Diagnosis Age**	57.08	59.73	58.42
**Sex**			
Female	533 (98.5%)	537 (99.3%)	1070 (98.9%)
Male	8 (1.5%)	4 (0.7%)	12 (1.1%)
**Race**			
Native American	1 (0.2%)	0 (0%)	1 (0.1%)
Asian	32 (5.9%)	28 (5.2%)	60 (5.5%)
African American	90 (16.6%)	92 (17.0%)	182 (16.8%)
White	379 (70.1%)	370 (68.4%)	749 (69.2%)
Unspecified	39 (7.2%)	51 (9.4%)	90 (8.3%)
**Cancer Type**			
Invasive Ductal Carcinoma	405 (74.9%)	375 (69.3%)	780 (72.1%)
Invasive Lobular Carcinoma	86 (15.9%)	115 (21.3%)	201 (18.6%)
Mixed Mucinous Carcinoma	4 (0.7%)	13 (2.4%)	17 (1.6%)
Metaplastic Breast Cancer	6 (1.1%)	2 (0.4%)	8 (0.7%)
Invasive Breast Cancer (NOS)	40 (7.4%)	36 (6.7%)	76 (7.0%)
**Cancer Subtype**			
Normal	19 (3.5%)	17 (3.1%)	36 (3.3%)
Luminal A	257 (47.5%)	242 (44.7%)	499 (46.1%)
Luminal B	69 (12.8%)	128 (23.7%)	197 (18.2%)
HER2	46 (8.5%)	32 (5.9%)	78 (7.2%)
Basal	99 (18.3%)	72 (13.3%)	171 (15.8%)
Unspecified	51 (9.4%)	50 (9.2%)	101 (9.3%)
**Stage**			
I	51 (9.4%)	38 (7.0%)	89 (8.2%)
IA	47 (8.7%)	39 (7.2%)	86 (7.9%)
IB	2 (0.4%)	3 (0.6%)	5 (0.5%)
II	3 (0.6%)	3 (0.6%)	6 (0.6%)
IIA	177 (32.7%)	178 (32.9%)	335 (31.0%)
IIB	123 (22.7%)	131 (24.2%)	254 (23.5%)
III	2 (0.4%)	0 (0%)	2 (0.2%)
IIIA	77 (14.2%)	78 (14.4%)	155 (14.3%)
IIIB	11 (2.0%)	17 (3.1%)	28 (2.6%)
IIIC	32 (5.9%)	32 (5.9%)	64 (5.9%)
IV	6 (1.1%)	13 (2.4%)	19 (1.8%)
X	10 (1.8%)	9 (1.7%)	19 (1.8%)

Supplementary Table 3: *Log-Rank p-values showing differences in overall patient survival between patients with high stromal and epithelial PXDN expression, high stromal and low epithelial PXDN expression, low stromal and high epithelial PXDN expression or low stromal and epithelial PXDN expression in tumours stained for PXDN by IHC in the CREA TMA cohort, corresponding to Figure 2G.*

	Epithelial high, stromal high	Epithelial high, stromal low	Epithelial low, stromal high
Epithelial high, stromal low	0.028		
Epithelial low, stromal high	0.01	<0.001	
Epithelial low, stromal low	0.75	0.11	0.1

Supplementary Table 4: *Demographics of patients in the CREA TMA invasive breast cancer cohort.*

**Total Patients**	334
**Average Diagnosis Age**	53.95
**Grade**	
1	45
2	109
3	155
Unclassified	25
**Molecular Subtype**	
Luminal A	191
Luminal B	41
HER2	26
Basal-like	52
Unclassified	24
**Treatment**	
None	57
Chemotherapy	57
Endocrine therapy	31
Both	66
Information Missing	123

Supplemental Information

Data S1/Method S1

Original R Code used in analysis

Analysis of PXDN association with overall patient survival in the TCGA Invasive Breast Cancer cohort

```
#Install the packages needed

library(readr)

library(ggplot2)

library(survminer)

library(readxl)

library(tidyverse)

library(data.table)

library(dplyr)

library(survival)

library(survminer)

library(ggpubr)


#Download the RNAseq data from TGCA

#Data used in this project was 2018
data_RNA_Seq_V2_mRNA_median_all_sample_Zscores

mRNA_Data <- readr(/data_RNA_Seq_V2_mRNA_median_all_sample_Zscores.txt)


#Re-structure the data to have a more readable format (Transpose rows and columns)

colnames(mRNA_Data)<- mRNA_Data[1,]

mRNA_Data <- subset(mRNA_Data, select = -Entrez_Gene_Id)

Transposed_mRNA_Data <- t(mRNA_Data)


#And re-name the column headings

colnames(Transposed_mRNA_Data)<- Transposed_mRNA_Data[1,]

Transposed_mRNA_Data <- Transposed_mRNA_Data[-1, ]
```

```
#Find PXDN sequences - Data was filtered to only contain columns with PXDN

PXDN_mRNA_Data <- Transposed_mRNA_Data[, c("Sample ID", "PXDN")]

PXDN_mRNA_Data <- as.data.frame(PXDN_mRNA_Data)


#Download the corresponding clinical data from TCGA

Clinical_Data <- read_csv(/brca_tcga_pan_can_atlas_2018_clinical_cata.csv)


#Change the patient ID column name in the mRNA dataset to match the same column in
the clinical dataset

PXDN_mRNA_Data <- setDT(PXDN_mRNA_Data, keep.rownames = TRUE) []

names(PXDN_mRNA_Data) [1] <- paste("Sample ID")


#Combine the two datasets

Final_Data <- dplyr::full_join(Clinical_Data, PXDN_mRNA_Data, by = "Sample ID")


#Survival Analysis of PXDN in breast cancer

#Stratify PXDN expression using median PXDN mRNA values

PXDN_Median <- median(Final_Data$PXDN, na.rm = TRUE)


#Draw a Kaplan-Meier plot of patient survival stratifying for high or low PXDN

Test <- Final_Data %>%

        mutate(Overall_Survival_Status_Modified = ifelse(`Overall Survival Status`==
"1:DECEASED", 2, 1)) %>%

        mutate(Median_Expression = ifelse(PXDN <= PXDN_Median, "Low", "High"))

fit_OS <- survfit(Surv(`Overall Survival (Months)`, `Overall_Survival_Status_Modified`) ~
Median_Expression, data = Test)

ggsurvplot(fit_OS, data = Test,

         surv.median.line = "hv",

         pval = TRUE,

         xlab = "Patient Overall Survival (Months)",

         ylab = "Survival Probability",
```

```
          font.x = c(15, "bold"),

          font.y = c(14, "bold"),

          font.legend = list(size = 14),

          legend = c(0.15,0.35),

          legend.labs = c("High PXDN", "Low PXDN"))


#Draw a Kaplan-Meier plot of patient survival stratifying for high or low PXDN as well as disease stage

names(Test)[names(Test) == "Neoplasm Disease Stage American Joint Committee on Cancer Code"] <- "Stage"

Test <- Test %>%

  mutate(stage_simplified = case_when(

    Stage %in% c("STAGE I", "STAGE IA", "STAGE IB") ~ "STAGE I",

    Stage %in% c("STAGE II", "STAGE IIA", "STAGE IIB") ~ "STAGE II",

    Stage %in% c("STAGE III", "STAGE IIIA", "STAGE IIIB", "STAGE IIIC") ~ "STAGE III",

    Stage %in% c("STAGE IV") ~ "STAGE IV", Stage %in% c("STAGE X") ~ "UNKNOWN",

    TRUE ~ as.character(Stage)

  ))


fit_Stage <- survfit(Surv(`Overall Survival (Months)`, `Overall_Survival_Status_Modified`) ~ Median_Expression + stage_simplified, data = Test)

plot_Stage <- ggsurvplot(fit_Stage, data = Test,

                          linetype = c("Median_Expression"),

                          color = c("stage_simplified"),

                         surv.median.line = "hv",

                         pval = TRUE,

                         xlab = "Patient Overall Survival (Months)",

                         ylab = "Survival Probability",

                         font.x = c(15, "bold"),

                         font.y = c(14, "bold"),

                         font.legend = list(size = 14),
```

```
                 legend = c(0.88,0.8))
print(plot_Stage)
```

#Seperate the data out into each stage and draw new Kaplan-Meier curves of patient overall survival stratifying for high or low PXDN within patients from each individual stage

```
#Stage 1
fit_Stage_I <- survfit(Surv(`Overall Survival (Months)`, `Overall_Survival_Status_Modified`) ~ Median_Expression, data = Test,
                subset = stage_simplified == "STAGE I")
plot_Stage_I <- ggsurvplot(fit_Stage_I, data = Test[Test$stage_simplified == "STAGE I", ],
                    pval = TRUE,
                    palette = c("#FF3333", "#FF3333"),
                    xlab = "Patient Overall Survival (Months)",
                    ylab = "Survival Probability",
                    surv.median.line = "hv",
                    font.x = c(15, "bold"),
                    font.y = c(14, "bold"),
                    font.legend = list(size = 14),
                    linetype = c("Median_Expression"))
print(plot_Stage_I)

#Stage 2
fit_Stage_II <- survfit(Surv(`Overall Survival (Months)`, `Overall_Survival_Status_Modified`) ~ Median_Expression, data = Test,
              subset = stage_simplified == "STAGE II")
plot_Stage_II <- ggsurvplot(fit_Stage_II, data = Test[Test$stage_simplified == "STAGE II", ],
                pval = TRUE,
                palette = c("#CC9900", "#CC9900"),
                xlab = "Patient Overall Survival (Months)",
                ylab = "Survival Probability",
```

```
                        surv.median.line = "hv",
                        font.x = c(15, "bold"),
                        font.y = c(14, "bold"),
                        font.legend = list(size = 14),
                        linetype = c("Median_Expression"))
print(plot_Stage_II)


#Stage III
fit_Stage_III <- survfit(Surv(`Overall Survival (Months)`, `Overall_Survival_Status_Modified`) ~
Median_Expression, data = Test,
                        subset = stage_simplified == "STAGE III")
plot_Stage_III <- ggsurvplot(fit_Stage_III, data = Test[Test$stage_simplified == "STAGE III",
],
                            pval = TRUE,
                            palette = c("#009966", "#009966"),
                            xlab = "Patient Overall Survival (Months)",
                            ylab = "Survival Probability",
                            surv.median.line = "hv",
                            font.x = c(15, "bold"),
                            font.y = c(14, "bold"),
                            font.legend = list(size = 14),
                            linetype = c("Median_Expression"))
print(plot_Stage_III)


#Stage IV
fit_Stage_IV <- survfit(Surv(`Overall Survival (Months)`, `Overall_Survival_Status_Modified`) ~
Median_Expression, data = Test,
                        subset = stage_simplified == "STAGE IV")
plot_Stage_IV <- ggsurvplot(fit_Stage_IV, data = Test[Test$stage_simplified == "STAGE IV",
],
                            pval = TRUE,
```

```
                    palette = c("#3366FF", "#3366FF"),
                    xlab = "Patient Overall Survival (Months)",
                    ylab = "Survival Probability",
                    surv.median.line = "hv",
                    font.x = c(15, "bold"),
                    font.y = c(14, "bold"),
                    font.legend = list(size = 14),
                    linetype = c("Median_Expression"))
print(plot_Stage_IV)
```

Analysis of PXDNs association with overall patient survival at the protein level in the CREA TMA cohort of breast cancer

```
# Load packages
library(ggplot2)
library(readxl)
library(dplyr)
library(survminer)
library(survival)
library(tidyr)
```

```
#Load file containing the scores for IHC

PXDN_CREA <- read_excel(/PXDN_CREA_Scoring_sheet.xlsx)


#Import clinical

Clinical_Data <- read_excel(/CREA_Clinical_Data.xlsx)


#Remove duplicate patient IDs, keeping the row with the highest grade tumours

Clinical_Data <- Clinical_Data %>% mutate(Grade = ifelse(`HistologicalGrade` == '1 - Low', 1, ifelse(`HistologicalGrade` == '2 - Medium', 2, ifelse(`HistologicalGrade` == '3 - High', 3, NA) )))

Clinical_Data <- Clinical_Data[order(Clinical_Data[,"PatientID"],-Clinical_Data[,"Grade"]),] #Order the duplicates

Clinical_Data = Clinical_Data[!duplicated(Clinical_Data$PatientID),]   #Remove smaller grade


#Tidy up the Patient Treatment Column formatting

Clinical_Data <- Clinical_Data %>% mutate(Treatment = ifelse(`Chemotherapy` == 1 & `Endocrine_therapy` == 1, "Both", ifelse(`Chemotherapy` == 1, "Chemo", ifelse(`Endocrine_therapy` == 1, "Endocrine", "None"))))


#Combine the IHC data with the clinical data

Final_df <- merge(PXDN_CREA, Clinical_Data, 'PatientID')


#Set up the data so that patients are not repeated (skewing the survival results)


#First create the censoring for KM plots based on patient survival (2 = patient still alive (censored event), 1 = patient died (event occured))

Final_df <- Final_df %>% mutate(`Survival_Censoring` = ifelse(`BrCaDeath` == 'Y', 2, 1))


#Combine multiple scores from the same patient based on the worst diagnosis and grade

#Using maximal H-scores

Max_dataset <- Final_df %>%
```

```
  group_by(`PatientID`, `Adjusted_Diagnosis`) %>%
  summarise(
    `TMA #` = first(`TMA #`),
    `Survival_Censoring` = first(`Survival_Censoring`),
    `Subtype` = first(`Subtype`),
    `Days_to_last_follow` = first(`Days_to_last_follow`),
    `BrCaDeath` = first(`BrCaDeath`),
    `Grade` = first(`Grade`),
    `Adj_chemo` = first(`Adj_chemo`),
    `Age` = first(`Age`),
    `Treatment` = first(`Treatment`),
    `Epithelial_H_score` = max(`Epithelial_H_score`),
    `Stromal_H_score` = max(`Stromal_H_score`),
    `Stromal_coverage` = max(`Stromal_coverage`),
    `Ki67_av` = first(`Ki67_av`),
    .groups = 'drop' )
#Reminder: add chemo


#The above code combines H-scores for cores from the same patient with the same diagnosis, but there are still duplicates in the PatientID where they had different diagnoses (E.g. IDC and DCIS)
#To remove these, I made a seperate column to give a numeric order to diagnoses, then kept only the highest diagnoses (e.g. IDC) for each patient
Max_dataset <- Max_dataset %>% mutate(`Diagnosis_Rank` = ifelse(`Adjusted_Diagnosis` == 'HEALTHY', 1,
                                         ifelse(`Adjusted_Diagnosis` == 'DCIS', 2,
                                              ifelse(`Adjusted_Diagnosis` == 'Ca', 3,
                                                   ifelse(`Adjusted_Diagnosis` == 'ILC', 4,
                                                        ifelse(`Adjusted_Diagnosis` == 'IDC', 5, 0))))))
Max_dataset <- Max_dataset %>%
  group_by(`PatientID`) %>%
```

```
  summarise(
    `Diagnosis_Rank` = max(`Diagnosis_Rank`),
    `Adjusted_Diagnosis` = first(`Adjusted_Diagnosis`),
    `Survival_Censoring` = first(`Survival_Censoring`),
    `TMA #` = first(`TMA #`),
    `Subtype` = first(`Subtype`),
    `Days_to_last_follow` = first(`Days_to_last_follow`),
    `BrCaDeath` = first(`BrCaDeath`),
    `Grade` = first(`Grade`),
    `Adj_chemo` = first(`Adj_chemo`),
    `Treatment` = first(`Treatment`),
    `Age` = first(`Age`),
    `Epithelial_H_score` = first(`Epithelial_H_score`),
    `Stromal_H_score` = first(`Stromal_H_score`),
    `Stromal_coverage` = first(`Stromal_coverage`),
    `Ki67_av` = first(`Ki67_av`),
    .groups = 'drop' )

#After this clean up step, there were too few patients with diagnoses other than IHC for statistical significance, so only IDC patients were included
Invasive_BC <- Max_dataset[Max_dataset$`Adjusted_Diagnosis` =="IDC",]

#Fix some of the NA rows
Invasive_BC$Subtype <- Invasive_BC$Subtype %>% replace_na("Unclassified")
Invasive_BC$Grade <- as.character(Invasive_BC$Grade)
Invasive_BC$Grade <- Invasive_BC$Grade %>% replace_na("Unclassified")

#The PXDN Allred scores were used to stratify patients into high and low stromal or epithelial PXDN groups using the mean Allred score for each compartment
#First the mean score was identified
mean(Invasive_BC$Stromal_H_score, na.rm = TRUE)
```

```
mean(Invasive_BC$Epithelial_H_score, na.rm = TRUE)

#Then high and low PXDN groups were created

Invasive_BC <- Invasive_BC %>% mutate(St_Rank = ifelse(Stromal_H_score > 4.2, "High",
"Low"))

Invasive_BC <- Invasive_BC %>% mutate(Ep_Rank = ifelse(Epithelial_H_score > 7.7,
"High", "Low"))


#Tests for independence were used to test for associations between PXDN and other
factors in the dataset

Chi_Ind <- chisq.test(table(Invasive_BC$Subtype, Invasive_BC$St_Rank))

Chi_Ind <- chisq.test(table(Invasive_BC$Subtype, Invasive_BC$Ep_Rank))

Chi_Ind <- chisq.test(table(Invasive_BC$Treatment, Invasive_BC$St_Rank))

Chi_Ind <- chisq.test(table(Invasive_BC$Treatment, Invasive_BC$Ep_Rank))

fisher.test(table(Invasive_BC$Grade, Invasive_BC$St_Rank))

fisher.test(table(Invasive_BC$Grade, Invasive_BC$Ep_Rank))


#Drawing Kaplan-Meier curves of patient overall survival stratified by epithelial PXDN

fit_Ep <- survfit(Surv(`Days_to_last_follow`, `Survival_Censoring`) ~ `Ep_Rank`, data =
Invasive_BC)

plot_Ep <- ggsurvplot(fit_Ep, data = Invasive_BC,

                size = 2,

                pval = TRUE,

                font.tickslab = c(15, "plain"),

                font.x = c(15, "bold"),

                font.y = c(14, "bold"),

                font.legend = list(size = 14),

                xlab = "Patient Overall Survival (Days)",

                ylab = "Survival Probability",

                legend = c(0.15, 0.38),

                legend.labs = c("PXDN High", "PXDN Low"))

print(plot_Ep)
```

#Drawing Kaplan-Meier curves of patient overall survival stratified by stromal PXDN

```
fit_St <- survfit(Surv(`Days_to_last_follow`, `Survival_Censoring`) ~ `St_Rank`, data =
Invasive_BC)

plot_St <- ggsurvplot(fit_St, data = Invasive_BC,
                      surv.median.line = "hv",
                      size = 2,
                      pval = TRUE,
                      font.tickslab = c(15, "plain"),
                      font.x = c(15, "bold"),
                      font.y = c(14, "bold"),
                      font.legend = list(size = 14),
                      legend = c(0.15,0.38),
                     legend.labs = c("PXDN High", "PXDN Low"),
                     xlab = "Patient Overall Survival (Days)",
                     ylab = "Survival Probability")

print(plot_St)
```

#Create a combined epithelial and stromal score

```
Invasive_BC <- Invasive_BC %>% mutate(Combined_Rank = ifelse(Ep_Rank == "High" &
St_Rank == "High", "EHSH",
                                      ifelse(Ep_Rank == "High" & St_Rank == "Low", "EHSL",
                                          ifelse(Ep_Rank == "Low" & St_Rank == "High", "ELSH",
                                              ifelse(Ep_Rank == "Low" & St_Rank == "Low",
"ELSL", "Errors")))))
```

#Drawing Kaplan-Meier curves of patient overall survival stratified by combined stromal and epithelial PXDN score

```
fit_comb <- survfit(Surv(`Days_to_last_follow`, `Survival_Censoring`) ~ `Combined_Rank`,
data = Invasive_BC)

plot_comb <- ggsurvplot(fit_comb, data = Invasive_BC,
                     surv.median.line = "hv",
```

```
                size = 2,
                font.tickslab = c(15, "plain"),
                font.x = c(15, "bold"),
                font.y = c(14, "bold"),
                font.legend = list(size = 14),
                legend = c(0.25,0.15),
                legend.labs = c("Epithelial High, Stromal High", "Epithelial High, Stromal Low","Epithelial Low, Stromal High","Epithelial Low, Stromal Low"),
                xlab = "Patient Overall Survival (Days)",
                ylab = "Survival Probability")
print(plot_comb)


#Exploring each variable in the dataset with univariate cox proportinal hazard tests
uni_cox_Age<-coxph(Surv(`Days_to_last_follow`, `Survival_Censoring`) ~ Age, Invasive_BC)
summary(uni_cox_Age)
uni_cox_Grade<-coxph(Surv(`Days_to_last_follow`, `Survival_Censoring`) ~ as.factor(Grade), Invasive_BC)
summary(uni_cox_Grade)
uni_cox_Stroma<-coxph(Surv(`Days_to_last_follow`, `Survival_Censoring`) ~ as.factor(Stromal_coverage), Invasive_BC)
summary(uni_cox_Stroma)
#Some groups need to be assigned levels first so that the correct group is chosen as a reference
Invasive_BC$Ep_Rank <- factor(Invasive_BC$Ep_Rank, levels = c("Low", "High"))
uni_cox_Ep<-coxph(Surv(`Days_to_last_follow`, `Survival_Censoring`) ~ as.factor(Ep_Rank), Invasive_BC)
summary(uni_cox_Ep)
Invasive_BC$St_Rank <- factor(Invasive_BC$St_Rank, levels = c("Low", "High"))
uni_cox_St<-coxph(Surv(`Days_to_last_follow`, `Survival_Censoring`) ~ as.factor(St_Rank), Invasive_BC)
summary(uni_cox_St)
```

```
Invasive_BC$Subtype <- factor(Invasive_BC$Subtype, levels = c("Luminal A", "Luminal B", "HER2", "Basal-Like", "Unclassified"))

uni_cox_Subtype<-coxph(Surv(`Days_to_last_follow`, `Survival_Censoring`) ~ as.factor(Subtype), Invasive_BC)

summary(uni_cox_Subtype)

Invasive_BC$Treatment <- factor(Invasive_BC$Treatment, levels = c("None", "Chemo", "Endocrine", "Both"))

uni_cox_Treatment<-coxph(Surv(`Days_to_last_follow`, `Survival_Censoring`) ~ as.factor(Treatment), Invasive_BC)

summary(uni_cox_Treatment)


#Grade, subtype, treatment, Ep-Rank and St_Rank all have effects on survival so were included in a multivariate cox proportional hazard model

res.cox <- coxph(Surv(`Days_to_last_follow`, `Survival_Censoring`) ~ Ep_Rank + St_Rank + as.factor(Grade) + Subtype +Treatment, data = Invasive_BC)

summary(res.cox)
```
